# In vitro production significantly reduces metabolic differences among bovine embryos

**DOI:** 10.1007/s11306-025-02352-x

**Published:** 2025-12-13

**Authors:** Denis Laloë, Julie Gatien, Camille Dupuy, Catherine Archilla, Ludivine Laffont, Sylvie Ruffini, Eugénie Canon, Daniel Le Bourhis, Marie-Christine Deloche, Olivier Dubois, Brigitte Leguienne, Lydie Nadal-Desbarats, Sophie Calderari, Clémentine Escouflaire, Olivier Desnoes, Laurent Schibler, Pascal Salvetti, V. Duranthon

**Affiliations:** 1 UMR1313 GABI, INRAE, Université Paris-Saclay, AgroParisTech, 78350 Jouy-en-Josas, France; 2 Eliance, 37380 Nouzilly, France; 3 Université Paris-Saclay, AP-HP, UVSQ, INRAE, BREED, 78350 Jouy-en-Josas, France; 4 Imaging Brain & Neuropsychiatry iBraiN U1253 , Université de Tours INSERM , 37032 Tours, France; 5 Eliance, 75012 Paris, France; 6 Ecole Nationale Vétérinaire d’Alfort, BREED, 94700 Maisons-Alfort, France; 7 Plateforme de Métabolomique et d’Analyses Chimiques, US-61 ASB, Université de Tours CHRU Tours, Inserm, Tours, France

**Keywords:** Metobolomics, Spent-culture-media, Sex, In-vivo-developed-embryos, In-vitro-produced-embryos, 1H-NMR spectroscopy

## Abstract

**Introduction:**

Although the in vitro production of bovine embryos now exceeds in vivo production, the quality of in vitro-produced embryos remains inferior. Metabolomic analysis of the spent culture medium used for embryos is considered a relevant source of markers for subsequent developmental capacity, genetic status and sex. However, little is known about the sources of variability in this metabolome and their respective significance.

**Objective:**

We compared bovine embryo spent culture media from in vivo developed (IVD) and in vitro produced (IVP) embryos, and analyzed how they varied with embryo stage, grade and sex.

**Methods:**

Embryos were produced in vitro under two different conditions: SOF medium supplemented with 1% fetal calf serum or IVF Bioscience media, or developed in vivo. They were recovered at Day-6 and cultured individually for 26 hours. The culture medium was analyzed using 1H-NMR spectroscopy, and the embryos were staged, graded and sexed.

**Results:**

In vitro production significantly reduced inter-embryo variability of the 24 metabolites measured. The mean variance of metabolite content was 5 to 10 times lower in IVP than in IVD embryos (depending on the IVP conditions). IVD embryo stages and grades at the start or end of the culture period contributed to 8% to 18% of variability in the media content, but only 1% of variability for IVP embryos. IVD embryo stage at the start of culture significantly impacted metabolite content. IVD embryo sex did not globally impact composition of the spent culture medium, but the kinetics of decrease of about half of the metabolites differed between male and female blastocysts.

**Conclusions:**

Standardization of the metabolic properties of IVP embryos invites new questions regarding the impact of embryo biotechnology. Differences in metabolism between male and female IVD embryos are highly transient during the morula-blastocyst stages.

**Supplementary Information:**

The online version contains supplementary material available at 10.1007/s11306-025-02352-x.

## Introduction

With more than one million bovine embryos being produced in vitro each year, this worldwide production has recently exceeded that of in vivo embryos (International Embryo Technology Society IETS, 2023). In vitro production in this species involves the in vitro maturation and fertilization of oocytes followed by a seven-day culture of the embryo, from the fertilized zygote to the blastocyst stage. It enables improvements to fertility in a few specific cases, such as heat stressed females or in the case of “repeat-breeder” cows that failed to establish pregnancy after artificial insemination (Hansen, 2020a). But above all it optimizes the use of females of high genetic value in selection schemes and therefore permits the more rapid dissemination of genetic progress. However, despite many attempts to optimize the conditions for in vitro embryo production, the quality of in vitro produced (IVP) embryos remains suboptimal and the pregnancy rates after transfer in recipient females remains about 10% lower for IVP embryos than for those developed in vivo (IVD) (Ealy et al., 2019; Hansen, 2020b). The search for non-invasive criteria predictive of the developmental ability of IVP embryos after their transfer in recipient females is therefore a priority concern in the field of bovine selection. Among the criteria studied, assessing morphological quality at different stages during the seven-day in vitro development period, or at the end of this period when embryos reach the blastocyst stage, remains the most frequently employed (Rabel et al., 2023). However, many studies tend to validate other criteria (Wrenzycki, 2021), including quantification of the different components in the embryo’s spent culture medium (Muñoz et al., 2014; Fernandes et al. 2021; Gimeno et al., 2021). Recent data nevertheless evidenced that many factors may influence the composition of this spent culture medium (Gimeno et al., 2022). However, these factors and their relative impact remain poorly documented. Furthermore, most metabolomic analyses have been conducted on IVP embryos. We therefore wondered about the extent to which metabolomic analysis of the spent culture media could detect differences between embryos according to their stage of development, morphological quality and sex, and if these differences are similar depending on whether the embryo has been developed in vivo or in vitro. Because our aim was to compare IVP and IVD embryos, we focused our analysis on spent culture media exposed to embryos for a short period of time (26 h) from Day-6 to Day-7 of development, so that IVD embryos recovered at the morula-blastocyst stage by the uterine flush could be analyzed. We therefore analyzed a large number (more than 1000 samples) of single spent culture media from IVP and IVD Holstein embryos. The embryos were phenotyped at the start and end of the culture period (26 h: from Day-6 to Day-7) by staging and grading according to IETS criteria, and were sexed by genotyping. Spent culture media were analyzed using 1 H-NMR spectroscopy as a robust quantitative method which does not require any previous sample preparation and is thus suitable for the large scale analysis of small fluid samples (Asampille et al., 2020).

## Materials and methods

All experimental protocols were carried out in accordance with European Directive 2010/63/EU on the protection of animals used for scientific purposes, and approved by the French Ministry of National Education, Higher Education, Research and Innovation after ethical assessment by the local ethics committee “ Comité d’Ethique en Expérimentation Animale Val de Loire” (protocol registered under APAFIS number 14205–2018032111305688 v2).

### Embryo and culture media production and characterization

In vivo embryo production. Embryos were obtained by the artificial insemination of 16 Holstein heifers at our experimental farm. The estrus of donor heifers was synchronized, and they were stimulated as described by Janati Idrissi et al. (2021). They underwent artificial insemination using semen from one of the 15 Holstein bulls selected to be the least related possible, so as to guarantee representation of all variants with a frequency > = 10% in the Holstein population and to retain the majority of alleles with a frequency of between 5 and 10%. Embryos were recovered by uterine flush, as described by Janati Idrissi et al. (2021), but 6.5 days after the first insemination.

In vitro embryo production. Oocytes were obtained from Holstein ovaries recovered from the slaughterhouse. Cumulus oocyte complexes (COCs) were aspirated from follicles 2–8 mm in diameter. Only grade 1 and 2 COCs (according to the International Embryo Technology Society (IETS) grading recommendations) were selected for maturation. In vitro maturation and in vitro fertilization (IVF) were performed as described by Janati Idrissi et al. (2021) except that epidermal growth factor was added at a rate of 10ng/ml to the maturation medium. The semen used for IVF came from the same bulls as those used for in vivo embryo production. After 22 h in the fertilization medium, cumulus cells were removed from presumptive zygotes by progressive vortexing for three minutes, and gentle pipetting. Day1 zygotes were then placed in Primovision™ 16-well Micro well group culture dishes (Ref #16606) and covered by 150µL Synthetic Oviductal Fluid (SOF, Minitüb, Gmbh, Germany) supplemented with 1% estrus cow serum, 2% MEM100 X, 1%BME 50 X, 0.33 g/L Na-Pyruvate and 6 g/L fatty acid free BSA (Sigma Aldrich #B6766, #M7145, #P2256 and #A6003 respectively). The culture medium was then covered with 3.7 mL of mineral oil (ORIGIO #10100500). Embryo development was monitored (one image every 15 min) over 5 days using the PrimoVision™ digital in vitro embryo development monitoring and archiving system.

Production of individual spent culture media. On Day 5, individual culture 4-well-plates were prepared and incubated at 38.5 °C under a maximal humidified atmosphere of 5%O_2_, 5%CO_2_ and 90% N_2_. Because our preliminary experiments had evidenced some inter-embryo contamination (probably through the mineral oil when several embryo-containing culture-droplets shared a common mineral oil layer), each embryo was cultured in a single well of a 4-wells plate. Three embryos were cultured per 4-well plate; the fourth well was dedicated to a control medium droplet devoid of any embryo. We also produced control medium in a single independent 4-well plate for each experiment.

146 h after the start of IVF, Day-6 in vitro produced morulae, early blastocysts and blastocysts were selected for a 26 h individual culture. Each selected embryo was graded and staged according to IETS (International Embryo Technology Society) recommendations (IETS Manual, 4th edition), rinsed twice in 350 µL SOF-BSA culture medium and individually transferred with 0.5 µL medium in a 22 µL SOF-BSA droplet under 650 µL Origio mineral oil prepared the day before in a 4-well culture plate. SOF-BSA medium was prepared from Synthetic Oviductal Fluid (SOF, Minitüb, Gmbh, Germany) supplemented with 2% MEM100 X, 1%BME 50 X, 0.33 g/L Na-Pyruvate and 6 g/L BSA (Sigma #A3311).

In vivo developed morulae and blastocysts were selected, rinsed twice in SOF-BSA and placed in individual culture, exactly as described for the in vitro produced embryos.

At the end of the 26-hour individual culture period (Day-7), 17 µl of spent culture medium was collected from each culture droplet and frozen immediately on dry ice before storage at −80 °C. Each embryo was also dry frozen for genotyping.

Embryo grading. At the beginning (Day 6) and end (Day 7) of the individual culture, the embryos were classified from Grade 1 to Grade 3 according to their decreasing morphological quality, as recommended by the International Embryo Transfer Society (IETS). Grade 1 embryos are of good or very good quality. They have a regular shape and uniformly coloured cells. Only a few cells are excluded from the embryo’s compact mass. Grade 2 embryos exhibit some irregularities in shape and colour, but at least 50% of the embryo remains intact. A few cells are excluded. Grade 3 embryos show cells of irregular size and non-uniform colour. Only around 25% of the embryo’s mass is intact, with a lot of material excluded from this mass.

In vitro embryo production in serum free media. Embryos were produced as described above, but in vitro maturation was performed in BO-IVM (IVF Biosciences), in vitro fertilization in BO-IVF and in vitro culture in Primovision™ 16-well Micro group culture plates in BO-IVC media. Between in vitro maturation and fertilization, in vitro fertilization and culture, and individual culture, oocytes/embryos were rinsed twice in Washing medium (IVF Biosciences) and twice in the following-step medium. Individual cultures were performed in SOF-BSA, as described above.

Embryo genotyping. Embryos were treated with proteinase K. Lysates were sent to Labogena for genotyping on the bovine EuroGMD array from Eurogenomics that contains 60,000 markers. The sexes of embryos were deduced from the genotyping results.

Embryo sexing. Embryos that failed to provide high quality genotyping results were sexed by PCR amplification of an Y-chromosome specific DNA sequence as described by Janati Idrissi et al. (2021).

### Metabolomics data/1 H-NMR spectroscopy results analysis

Samples of embryonic culture medium were analyzed by 1 H-NMR spectroscopy. The samples were prepared using 15 µL culture medium diluted with 184.5 µL 0.2 M potassium phosphate buffer in deuterium oxide (D_2_O) (pH 7.4 ± 0.5) and 1.5 µL 3-(Trimethylsilyl) Propionic-Acid solution at 3.2 mM, used as a spectrometer field lock signal. The resulting solution was then transferred to conventional 3-mm 1 H-NMR spectroscopy tubes. 1 H-NMR spectra were recorded at 298 K on a Bruker Ascend Avance III HD 600 MHz system (Bruker, Sadis, Wissembourg, France), equipped with a Bruker 5 mm TCI cryoprobe with Z-gradient. 1 H-NMR spectra were recorded using a “cpmgpr1d” pulse sequence with a 90° pulse, a relaxation delay of 25 s, and 256 scans on a time domain of 64 K data points. Data were processed with 0.2 Hz of line broadening for the exponential decay function using TopSpin version 3.6 software (Bruker Daltonik, Karlshure, Germany). Metabolite quantifications using the ERETIC peak as a quantitative reference were obtained by the specific subroutine of the Bruker TopSpin 3.6 program. The ERETIC signal was calibrated on an amino acid solution of a known concentration ([ERETIC] = 67 µM). Spectral assignments were done using the free version of ChenomX 7.1 software (ChenomX, Edmonton, Canada), an in-house database and a human metabolome data base (HMDB version 4.0; (HMDB version 4.0; http://www.hmdb.ca).

Metabolomic data normalization. For each metabolite in each embryo spent culture medium, the normalized value was obtained by subtracting from the embryo-medium raw value the mean of raw values obtained for all control media generated during the same experiment.

### Data analyses and visualizations

To analyze the metabolomic data and their relationships with experimental variables (development stages at Day-6 and Day-7, embryo grade at Day-6 and Day-7, sex), various data analyses were performed with R software (version 4.4.2, R Core Team, 2024). Multivariate factorial methods were performed first, as follows:

#### Multivariate factorial analyses

##### Principal component analysis (PCA)

PCA was first applied to the metabolomics data. This unsupervised method transforms the original data into a set of orthogonal components, ranked by the amount of variance they explain. PCA was used to assess the overall structure of the data, identify patterns, and visualize sample clustering. These analyses were performed using the ade4 package (Thioulouse et al. 2018).

##### Redundancy analysis (RDA)

RDA is an extension of PCA (Legendre and Legendre, 2012), that explicitly models a set of response variables as a linear function of explanatory variables. It was used to quantify the relationship between metabolome and experimental variables (sex, Day-6 and Day-7 stages and grades). The significance of the different factors was assessed through permutation tests (*n* = 1000 permutations). The analysis was performed using the vegan package in R (Oksanen et al., 2024).

##### Multiple correspondence analysis (MCA)

MCA (Abdi et al. 2007) was employed to explore relationships between the different experimental variables (sex, Day-6 and Day-7 Stages and Grades). MCA can be regarded as an adaptation of PCA to categorical data. The analysis was carried out using the ade4 package in R (Thioulouse et al. 2018). As in PCA, MCA provides a factorial map which describes a set of categorical variables according to the intensity and level of association between them. For better readability, this representation can be split by categorical variables.

Graphics corresponding to these multivariate analyses were generated using the adegraphics R package (Thioulouse et al. 2018).

#### Visualization of relationships between pairs of factors

In order to enhance the understanding of relationships between the different factors (sex, development stages and embryo grades), different types of plots were drawn:

##### Alluvial plots

Relationships between development stages at Day-6 and Day-7 were visualized using alluvial plots. These plots were generated with the ‘alluvial’ R package (Bojanowski & Edwards, 2016), which could illustrate how the distribution of development stages or embryo grades might change between Day-6 and Day-7. In an alluvial plot, categorical variables are displayed as parallel vertical axes, with flows (or “alluvia”) connecting them to reflect transitions between categories. The width of each flow is proportional to the count or proportion of observations in that category combination, allowing for the identification of patterns, trends and dependencies between categorical variables.

##### Mosaic plots

Mosaic plots were used to visualize contingency tables (Zeileis et al., 2007). This graphical representation is used to display the relationship between two factors. Each factor’s modalities are represented by tiles, with the area of each tile proportional to the frequency or count of observations in the corresponding category combination. The independence between the factors was tested with Fisher’s exact test (Agresti, 2010) and the corresponding p-value was added to the mosaic plot.

#### Univariate linear models

Univariate analyses of variance were performed for each metabolite. Moreover, because development stages have a natural ordering, we also evaluated whether the metabolites changed monotonically across the different development stages by testing for a linear trend according to stages, where stages were the ordered factors (Agresti, 2010).

## Results

Fifteen Holstein bulls, selected as described in Materials and Methods, were used for IVP embryo production, and IVD embryos were obtained from 11 of them. The embryos were cultured separately in 22 µl microdroplets covered by mineral oil, each culture droplet being placed in one well of a 4-well plate, thus preventing any inter-droplet contamination.

A total of 1438 samples were analyzed by 1 H-NMR spectroscopy, 371 of which were control media and 1067 embryo spent culture media. After data normalization (see Materials and Methods) and removal of outliers, our statistical analyses started on 851 embryo spent culture media, 90 from in vivo developed (IVD) embryos and 761 from in vitro produced (IVP) embryos.

The distribution of these IVD and IVP embryos between the stage and grade modalities at Day-6 (start of the 26-hour individual culture period) and Day-7 (end of the individual culture) are shown in Suppl. Table 1).

### Embryo development and grade after IVD or IVP

Embryos were staged according to IETS reference criteria at both Day-6 and Day-7. It should be noted that during the 26-hour individual culture period, 220 (22%) of the IVP embryos degenerated, and were thus not graded nor staged at Day-7. They were not considered in the 761 IVP embryos analyzed here. This situation was not observed for IVD embryos.

At both times (Day-6 and Day-7), developmental stages differed statistically at a 5% level between IVD and IVP embryos (Suppl. Fig. 1a and b), but the difference was more pronounced at Day-7 (Fisher test p-values: *p* = 0.048 and *p* < 0.01, respectively). At Day-7 in particular, the proportion of hatched and expanded blastocysts was higher in IVD embryos than in IVP embryos (Suppl. Fig. 1a, b). Analysis of the development kinetics between Day-6 and Day-7 further evidenced differences between IVP and IVD embryos. Notably, few IVP morulae did not evolve towards the blastocyst stage but all the IVD morulae did so. The proportions of Day-6 morulae, early blastocysts, blastocysts and expanded blastocysts that hatched between Day-6 and Day-7 was higher in IVD than in IVP embryos (Suppl. Fig. 1c).

Embryos were graded according to IETS references both at the start (Day-6) and end (Day-7) of the 26-hour individual culture period. Whatever the day, IVD and IVP embryo grades were statistically different (Fisher test p-values < 0.01) with nearly none of grade G3 and a higher proportion of grade G1 among IVD embryos (Suppl. Figure 2a, b). Also, the analysis of grade evolution between Day-6 and Day-7 showed a greater stability in grade between Day-6 and Day-7 in IVD embryos than in IVP embryos. In particular, a higher proportion of G2 embryos evolved toward grade G3 at Day-7 among IVP embryos than among IVD embryos (Suppl. Figure 2c).

It should be noted that multiple correspondence analysis of the embryos evidenced a link between staging and grading at Day-6 or Day-7, with the most advanced embryos in terms of stage displaying the best grade, whatever the embryo type (IVD or IVP) (Suppl. Figure 3).

### Impact of embryo stage and grade on the metabolome of spent culture media

Under our experimental conditions, we were able to quantify 24 metabolites that included 14 amino acids: alanine, asparagine, aspartate, glycine, histidine, isoleucine, leucine, lysine, methionine, phenylalanine, proline, threonine, tyrosine and valine; two carbohydrates: pyruvate and lactate; one ketonic compound (3-hydroxybutyrate); one fatty acid: 2-hybroxybutyrate, and six other organic compounds.

**Fig. 1:**
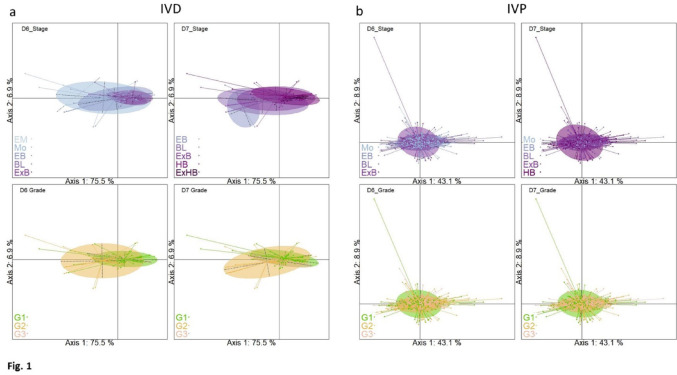
Spent culture medium of embryos: Principal Components Analysis (PCA) score plots. X-axis and Y-axis show the first and second principal component with the percentage of explained variance respectively. Embryos are clustered and colored according to their status (embryo stage or grade), and the ellipses represent the 95% confidence interval. Top left: Day-6 stage; Top right: Day-7 stage; Bottom left: Day-6 grade; Bottom right: Day-7 grade. (EM: Early morula, Mo: Morula, EB: Early Blastocyst, BL: Blastocyst, ExB: Expanded Blastocyst, HB: Hatched Blastocyst, ExHB: expanded hatched blastocyst, G1, G2, G3: Grade 1,2,3). **a** Spent culture medium of IVD embryos. **b** Spent culture medium of IVP embryos

Principal component analyses of the metabolomic results on spent culture media from IVD embryos showed that the embryo stage and grade at Day-6 and Day-7 impacted the spent culture medium (Fig. 1a). Redundancy analysis evidenced that Day-6 stage, Day-6 grade and Day-7 stages were respectively responsible for 18%, 13% and 14% of overall variability, while Day-7 grade explained only 8% of this variability (Table 1). The relative contributions of the metabolites varied depending on stages and grades. Asparagine contributed to variations whatever the stage and grade, acetylcholine appeared to be one of the most variable components with Day-6 grade and both Day-7 stage and grade. Further, methionine varied in spent culture media with Day-7 stage and with embryo grade at both Day-6 and Day-7. Finally, alanine appeared to be one of the most variable compounds, with both Day-6 grade and stage (Suppl. Fig.4).

**Table 1:** Redundancy analysis of metabolomic data of IVD and IVP embryos’ spent culture media according to Day-6 and Day-7 stages and grades

Variable	Inertia ratios IVD	*p*-values IVD	Inertia ratios IVP
D6-Stage	0.18	0.003	0.005
D6-Grade	0.13	0.003	0.003
D7-Stage	0.14	0.010	0.010
D7-Grade	0.08	0.014	0.004

Inertia ratios and associated p-values

When the spent culture media from IVP embryos was analyzed in the same way, no such variability was found. First, neither the stage of embryos nor their grade at either Day-6 or Day-7 could account for variability in the composition of the culture medium. Each variable made a very small contribution (1% for Day-7 stage and even less for Day-6 stage and Day-6 or Day-7 grades) to metabolome variability in the spent culture media (Table 1). This was also shown by PCA analyses which failed to separate IVP embryos according to their stage or grade (Fig. 1b). Moreover, among the 24 metabolites quantified, only two (acetylcholine and hydroxb3) were more variable in IVP spent culture media than in their IVD counterparts. For all other metabolites, the variances were 2 to 110 times lower in IVP than in IVD embryo spent culture media (Table 2).

**Table 2:** Comparison of metabolite variances according to embryo type (IVD and IVP)

Metabolite	IVD	IVP	Ratio IVD/IVP
2-hydroxybutyrate	377.57	85.18	4.43***
2-hydroxyisobutyrate	42.75	1.53	27.91***
3-hydroxybutyrate	44.15	2557.98	0.02***
5,6-dihydrothymine	1.60	0.45	3.54***
Acetate	2249.33	20.14	111.66***
Acetylcholine	0.17	2.89	0.06***
Alanine	124.80	10.75	11.61***
Asparagine	8571.04	1302.28	6.58***
Aspartate	2199.76	275.81	7.98***
Formate	3956.53	82.08	48.2***
Glycine	1430.45	138.81	10.31***
Histidine	138.81	74.15	1.87***
Isoleucine	139.89	14.25	9.82***
Lactate	15899.58	1500.65	10.6***
Leucine	104.86	8.37	12.53***
Lysine	616.08	65.82	9.36***
Methionine	144.67	28.28	5.12***
Phenylalanine	146.51	21.17	6.92***
Proline	42.41	19.04	2.23***
Pyroglutamate	1565.67	294.46	5.32***
Pyruvate	132096.63	61986.77	2.13***
Threonine	591.00	176.83	3.34***
Tyrosine	128.93	17.97	7.18***
Valine	408.32	74.47	5.48***
Mean	7125.90	2865.01	13.09

In columns 2 and 3, variances; in column 4: variance ratios between the two conditions

The number of stars indicates the level of statistical significance of the corresponding Levene test for variance homogeneity: ***P<0.01, **P<0.05, *P<0.10

### Impact of embryo sex on spent culture media

We then addressed the impact of the sex of embryos on the metabolome of spent culture media under our experimental conditions. We therefore sexed the IVD and IVP embryos by genotyping. For those whose call rate was not sufficient to conclude, we performed PCR using primers corresponding to the Y chromosome sequence. We succeeded in sexing 80 IVD and 540 IVP embryos and further analyzed them.

We studied whether embryo sex had any effect on stage or grade in IVD and IVP embryos. There was no difference between male and female embryos regarding stage at Day-6 or Day-7, irrespective of whether we considered all embryos, IVD or IVP embryos (Suppl. Fig. 5). By contrast, for IVD embryos only, a difference appeared with embryo sex in terms of its grade at Day-6 and Day-7, with a higher proportion of male grade 1 embryos than female grade 1 embryos (Suppl. Tables 2 and Suppl. Figure 6).

**Fig. 2:**
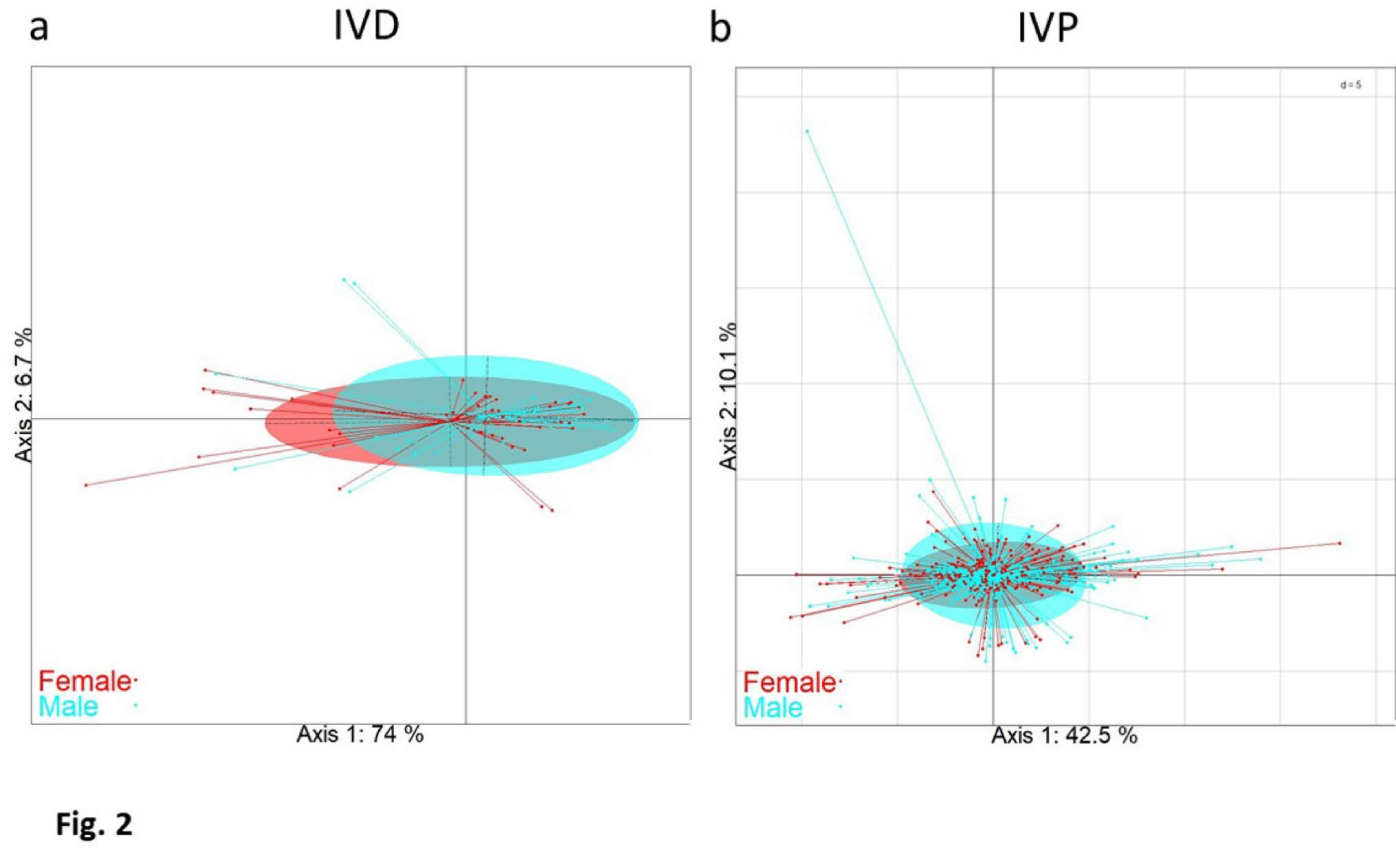
Spent culture medium of IVD (**a**) and IVP (**b**) embryos: Principal Components Analysis (PCA) score plots. X-axis and Y-axis show the first and second principal component with the percentage of explained variance respectively. Embryos are clustered and colored according to their sex, and the ellipses represent the 95% confidence interval

We next analyzed the metabolomes of spent culture media as a function of embryo sex. PCA analysis of their metabolomic features was not able to separate the embryos according to their sex (Fig. 2a, b). This was corroborated by RDA modelling of the metabolome according to sex, and a Monte-Carlo test with 999 replicates: p-val = 0.253 and 0.754 for IVD and IVP embryos, respectively. Moreover, redundancy analyses including stage, sex and their interaction or grade, sex and their interaction at Day-6 or Day-7, for either IVD or IVP embryos, failed to evidence any significant sex effect at a 5% level, but pointed to a significant sex*stage interaction at Day-7 for IVD embryos (p-val = 0.014) (Suppl. Table 3).

**Fig. 3:**
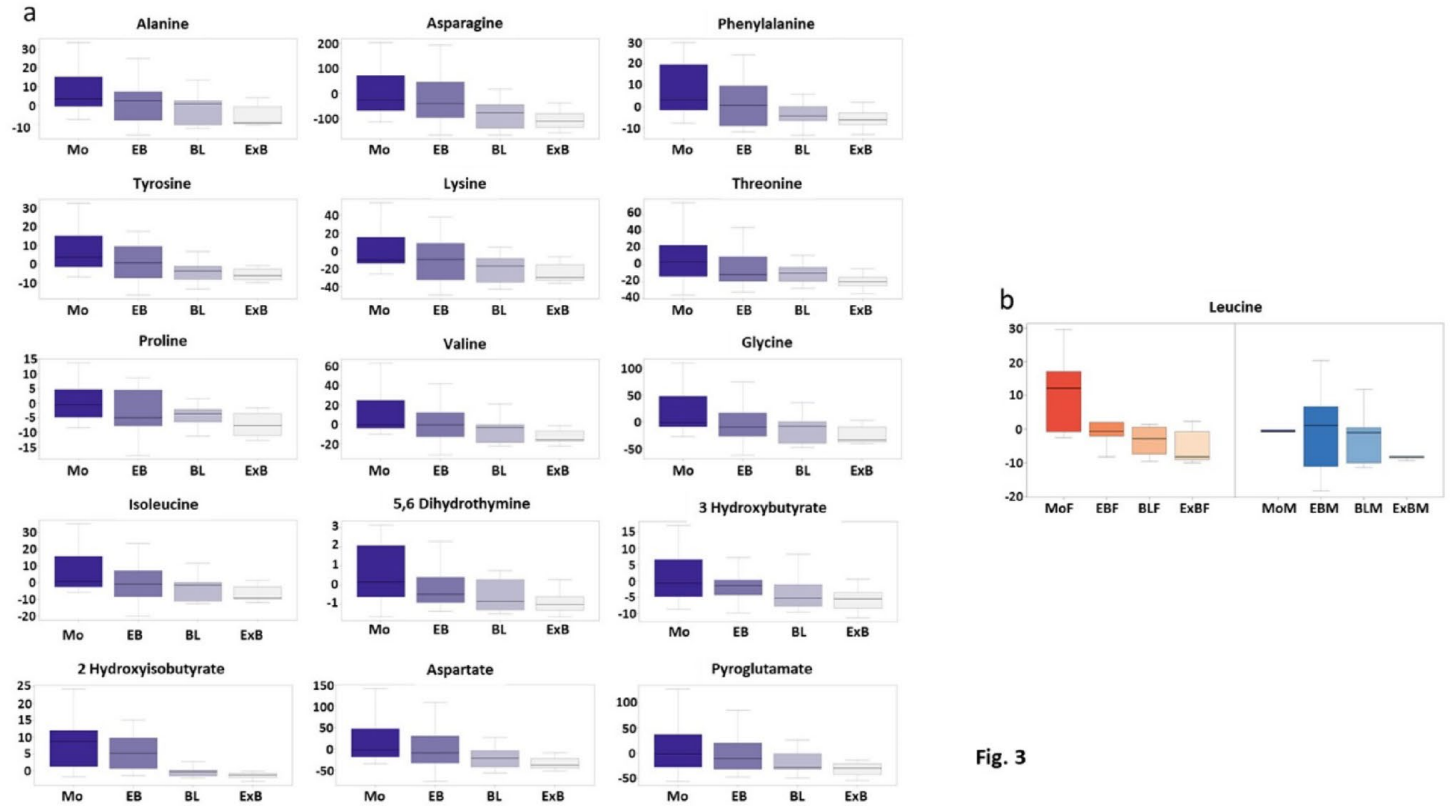
Quantification of metabolites with the most significant Day-6 stage effect (see also Suppl Table 4 A) (**a**) or a significant Sex*Day-6 stage interaction (**b**) in spent culture media according to the Day-6 stage of IVD embryos. For each metabolite and for each stage plotted on the X-axis, the net balance of extracellular metabolites released or consumed by embryos is plotted on the Y-axis. *Mo* morula, *EB* early blastocyst, *BL* blastocyst, *ExB* expanded blastocyst, *F* female, *M* male

Then, in order to analyze in greater depth the variability of spent culture media for IVD embryos, we used linear models per permutations with stage, sex, and stage*sex interaction effects. In particular, to prevent any bias, we removed from our further analyses two embryos at the early morula stage on Day-6, because they represented a very scarce Day-6 stage and both were females. Most of the metabolites displayed a significant Day-6 stage effect (p-value < 0.05). For all the amino acids except histidine, the more advanced the embryo was at the start of the individual culture, the lower the quantity of amino acids was found in the spent culture medium (Suppl. Table 4A, Fig. 3a). Moreover, Anova for ordered factors was used because the developmental stages were ordered factors. For all the metabolites quantified, we evidenced a significant linear trend (Suppl. Table 4B), thus showing a monotonic decrease of metabolomic variables according to the developmental Day-6 stage. In addition, leucine also displayed a significant sex effect (see Suppl. Table 4A and Fig. 3b).

**Fig. 4:**
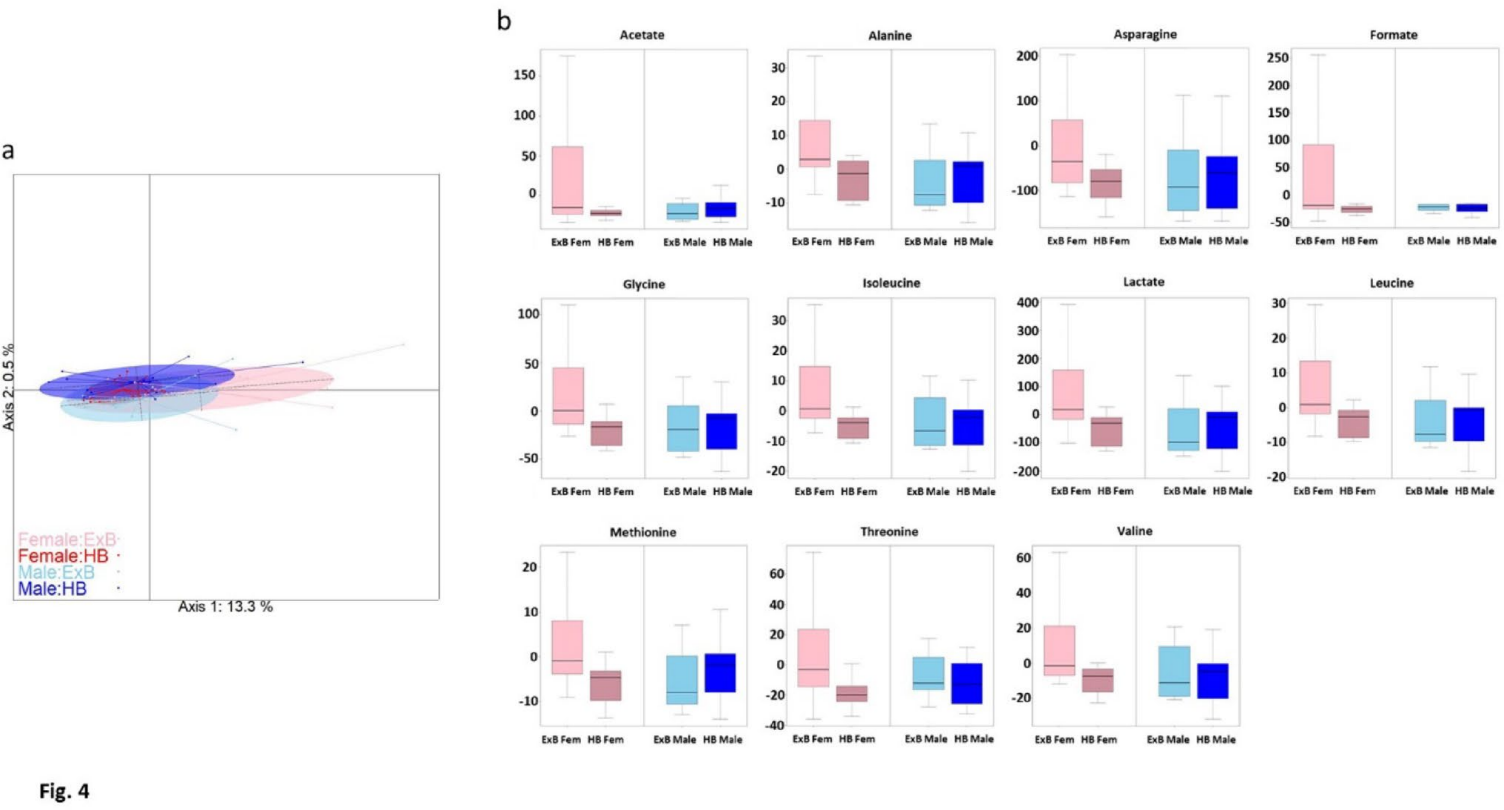
Analysis of the Sex*Day-7 stage interaction: **a** Principal Components Analysis (PCA) score plot. X-axis and Y-axis show the first and second principal component with the percentage of explained variance respectively. Embryos are clustered and colored according to embryo sex and Day-7 stage. **b** Quantification of metabolites with a significant Sex*Day-7 stage interaction in the spent culture media of male and female IVD embryos reaching the expanded or hatched blastocyst stage at Day-7. For each metabolite and for each embryonic Day-7 stage and sex plotted on the X-axis, the net balance of extracellular metabolites released or consumed by embryos is plotted on the Y-axis. *ExB* expanded blastocyst, *HB* hatched blastocyst, *Fem* female

The impact of the embryo Day-7 stage on spent culture media was then analyzed. To improve its robustness, we focused on the two most frequent Day-7 stages: expanded and hatched blastocysts. We thus analyzed 32 expanded blastocysts (16 males and 16 females) and 32 hatched blastocysts (17 males and 15 females). Principal component analysis evidenced a higher variability among female expanded blastocysts than among any other stage-sex group (Fig. 4a) (Monte-Carlo test with 999 replicates provided a significant p-value of 0.012). The analysis of individual metabolites using a linear model again evidenced a significant sex*stage interaction for 11 metabolites, including eight amino acids and lactate. The quantities of these metabolites decreased in the culture media of female embryos between the expanded and hatched blastocyst stages, but remained stable in the culture media of male embryos (Fig. 4b).

Additionally, a significant stage effect was observed at Day-7 for hydoxyb3, acetylcholine, lysine, proline, pyroglutamate and pyruvate (Suppl. Table 5; Suppl. Fig. 7).

### Less variability in embryo metabolism following in vitro production was also observed in serum-free IVP conditions

Our results evidenced a far less variable composition of spent culture media when the embryos had been produced in vitro, reflecting less variable metabolic activity in IVP embryos than IVD embryos. To determine whether this reduction in variability was dependent on the IVP conditions, we analyzed the spent culture media of IVP embryos obtained under different conditions and particularly without bovine calf serum in the media.

We thus produced embryos using IVF Bioscience media for in vitro oocyte maturation, in vitro fertilization and embryo culture until Day-6. Under these new IVP conditions, we still saw that 22% of the embryos degenerated during the individual culture period. Here again, their spent culture media were not analyzed. We reasoned that a hundred embryos was sufficient to evidence variability among IVD embryos, and therefore produced slightly more (176) embryo spent culture media under the new IVP conditions and analyzed them as previously. The kinetics of IVP embryo development between fertilization and Day-6 were slower under these new conditions, with fewer embryos reaching the blastocyst stage by Day-6, but more G1 and G2 embryos by Days 6 and 7 (Suppl. Tables 6a and 6b). By Day-6, 83% of the embryos had reached the morula stage. Of these, 75% progressed to the blastocyst stage (including hatched blastocysts) by Day-7 (see Suppl. Fig. 8).

**Fig. 5:**
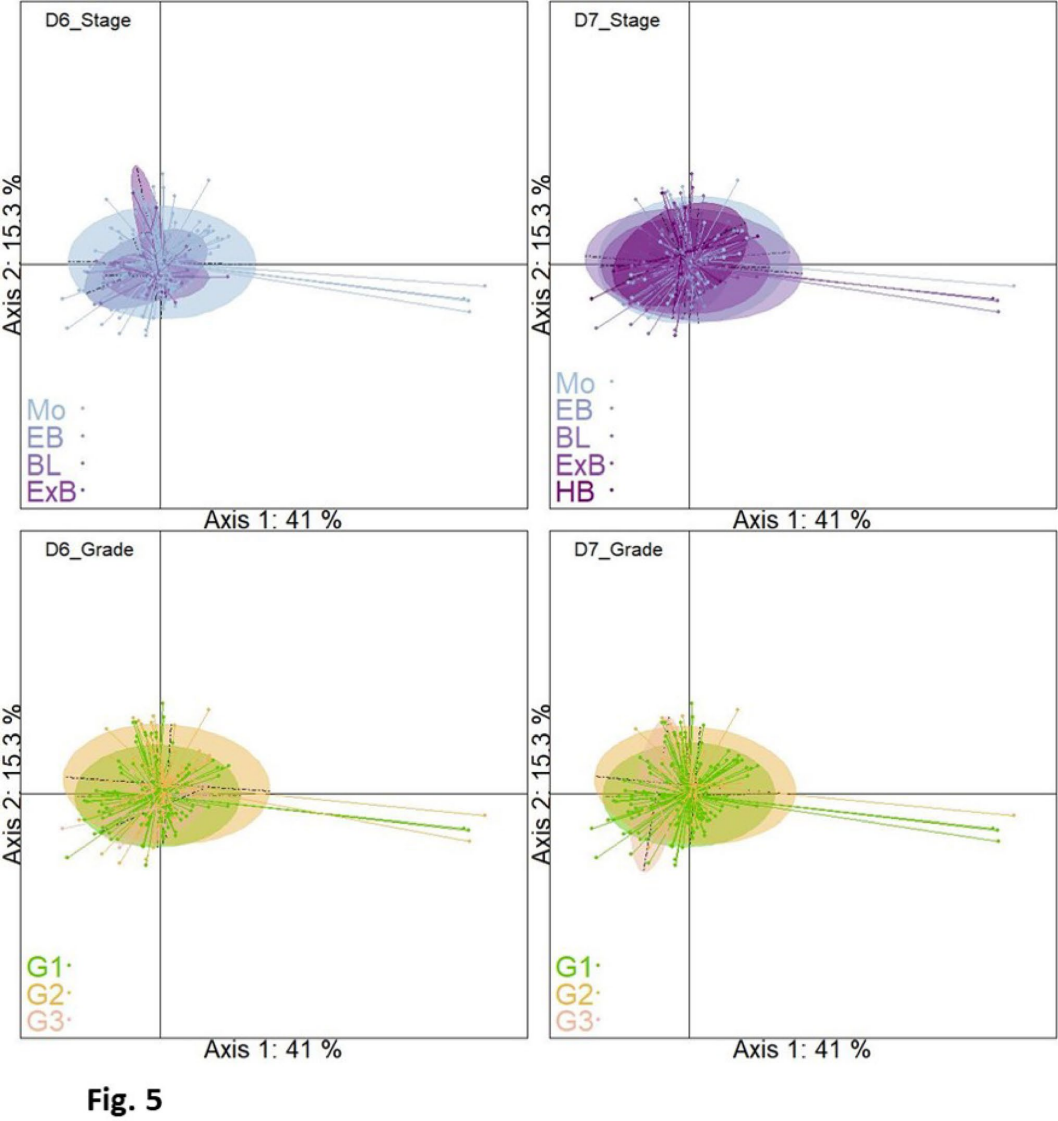
Spent culture medium of IVP embryos under IVF Bioscience conditions: Principal Components Analysis (PCA) score plots. X-axis and Y-axis show the first and second principal component with the percentage of explained variance respectively. Embryos are clustered and colored according to their status (embryo stage or grade), and the ellipses represent the 95% confidence interval. Top left: Day-6 stage; Top right: Day-7 stage; Bottom left: Day-6 grade; Bottom right: Day-7 grade. *Mo* morula, *EB* early blastocyst, *BL* blastocyst, *ExB* expanded blastocyst, *HB* hatched blastocyst, *ExHB* expanded hatched blastocyst, *G1*, *G2*, *G3* Grade 1,2,3

As for embryos produced in SOF serum, principal component analyses of the metabolome of spent culture media from embryos produced in IVF Bioscience media did not distinguish embryos according to their stage or grade at Day-6 or Day-7 (Fig. 5). Inertia ratios at Day-6 or Day-7 were between 1.1% and 1.5%. Also, redundancy analysis exploring sex and grade or sex and stage effects and their interactions failed to evidence any significant effect at Day-6 and only pointed to a significant sex*grade interaction at Day-7 (Suppl. Table 7).

Moreover, despite a significant overall increase in the variance of individual metabolites (mean 2.1-fold) compared to the SOF serum in vitro conditions as shown by the Levene test, most of the metabolites still displayed a much reduced variance compared to the IVD conditions (Suppl. Table 8).

We therefore concluded that the variability in embryo metabolism evidenced among IVD embryos was significantly reduced by in vitro production.

## Discussion

Embryo spent culture medium is regarded as a relevant and non-invasive source of markers for the further developmental ability, genetic status, or sex of embryos, and a variety of molecular markers are sought such as cell free DNA (Layek et al. 2024), miRNAs (Rio et al. 2024), and metabolites (Salmeron et al. 2021). In cattle, the metabolomic analysis of embryo culture media has been used to look for markers predictive of embryo capacity for post-transfer development (Muñoz et al. 2014; Perkel et Madan 2017; Fernandes et al. 2021) and also to predict embryo sex (Sturmey et al. 2010; E. Gómez et al. 2016; Rubessa et al. 2018; Gimeno et al. 2022), but their results were dependent on both embryo stage and the method of analysis, so no consensus has yet been reached. In this context, the originality of our study lay firstly in our comparison of IVP embryos with their IVD counterparts. It was also based on the choice not to whether the culture media metabolome enabled discrimination between embryos according to this or that factor, but to investigate how much of the its variability could be explained by the criteria of interest for those handling embryos (e.g. stage, grade or sex). Finally, the large number of embryos studied was also a major strength of our work.

In this context, our most striking result was the significant reduction in the variability of embryo spent culture media when the embryos were produced in vitro rather than developed in vivo, reflecting a major decrease in the variability of embryo metabolic activity. This finding appears to contradict the results obtained by Sturmey et al. (2010), who found greater variability in metabolic activity in IVP embryos. However, there are many differences between the two studies, which makes comparison difficult. Firstly, Sturmey et al. began individual culture one day later (Day-7 vs. Day-6). Also, their IVD population was smaller (*n* = 35 vs. 90) and recovered from slaughtered heifers. Furthermore, their IVD embryos were all grade 1 blastocysts at the start of individual culture. The individual culture conditions also differed: their embryos had the resources available in 4 µL of culture medium, compared with 22 µL in our study. All of these factors can affect the variability of IVD embryos.

In our study, the variance of most metabolites fell by 5 to 10-fold in the media of IVP embryos. Moreover, embryo stage and grade at the start and end of individual culture, which explained 13% to 18% of the variability in the culture medium composition of IVD embryos, explained less than 1% of variability for IVP embryos. Why was this? Whether IVD embryos already have different metabolic activities in utero remains unknown. In particular, molecular cross-talk between the embryo and the maternal tract (oviduct and/or uterus) during development could enhance metabolic variability. It has been demonstrated that the bovine endometrium responds differently to male and female embryos, and that uterine fluid contains different quantities of several proteins depending on the sex of the embryo (Gómez et al., 2013). Thus, as the differential response of the embryo to maternal embryokines has been suggested to be responsible for further sex-dependent programming (Hansen et Tríbulo 2019), differences in embryo metabolic activity may have emerged as responses to maternal signals. Such responses would partly depend on the stage of development and grade of the embryo but also to any other individual feature.

Conversely, we cannot formally exclude the possibility that IVD embryos were highly similar upon recovery from the uterus. In this case, variability in their spent culture media could reflect variability in their adaptation process to their new environment during the 26 h of in vitro culture. Indeed, the change in environmental conditions encountered by IVP embryos from their group (fertilization to Day-6) to their individual (from Day-6 to Day-7) culture conditions was probably far less. Nevertheless, because expected features such as embryo stage and sex impact IVD spent culture media, this was probably not the main reason for the variability of IVD embryos.

Another non-exclusive explanation is that group culture from fertilization to Day-6 had standardized the metabolic activity of IVP embryos, so they no longer differed from each other when cultured individually. The recent development of individual culture protocols for IVP embryos throughout the entire production period will soon enable experimental confirmation or negation of this hypothesis. However, group culture has been reported to modify the spent culture media of human embryos (Santos et al., 2019).

Finally, the composition of the media used for the in vitro production of embryos (because they are too rich in metabolites or too standardized compared to oviductal and uterine fluids) may drastically reduce variability in the metabolic activity of IVP embryos. Notably, we initially assumed that because of the nutrient richness of fetal calf serum, it might have been responsible for this abolition of variability, but a decrease in metabolomic variability was also observed in embryos produced using serum-free media (IVF Bioscience media). Despite the presence of a synthetic serum replacement in these media, it is probable that they are less complex in composition than SOF medium with added serum. Importantly, the decrease in metabolic variability was less pronounced in this new medium. This indicates that IVP conditions are partly responsible for this phenomenon. Furthermore, such media are increasingly used by professionals in the field, so it was important to confirm that our observations remained valid when using such media for the production of embryos in vitro.

It is worth noting that despite the markedly reduced variability seen in IVP spent culture media statistically significant effects were detectable for some metabolites using univariate analyses (data not shown). However, because these effects were so tiny, we choose not to pursue their analysis.

Regarding IVD embryo media, both redundancy and univariate analyses of individual metabolites evidenced a significant and linear effect for the Day-6 embryo stage. The more advanced the embryo was in its development at the start of the culture, the higher was its metabolite consumption. This probably reflected an increase in embryonic metabolic needs as the cell numbers increased and the first differentiation occurred. It is well known that embryo metabolism varies considerably around blastulation in order to cover energy demands for cavitation and differentiation (Milazzotto et al., 2020). As far as amino acids are concerned, such an increase of consumption has already been reported during bovine embryo development (Partridge & Leese, 1996). This increased consumption of exogenous compounds may also reflect a depletion of the embryo’s endogenous reserves which result from the degradation of maternal proteins during earlier stages of development (Tsukamoto et Tatsumi 2018).

Redundancy and univariate analyses also pointed to a significant sex*Day-7-stage interaction for 11 of the 24 metabolites analyzed. Several analyses of embryo spent culture media evidenced differences between male and female embryos, but no consensus can be reached on the metabolites involved (Sturmey et al. 2010; E. Gómez et al. 2016; Gomez et al. 2018; Rubessa et al. 2018; Gimeno et al. 2022). Most of these studies focused on culture media from embryos at a single stage i.e. blastocyst or expanded blastocyst (Sturmey et al. 2010), or from embryos evolving along a single developmental trajectory (for example Day-6 morula to Day-7 expanded blastocyst in Gómez et al. (2016). A sex effect in these previous studies may therefore have appeared as a sex*stage interaction in our experimental design that involved various stages and trajectories. Analyses by Sturmey et al. (2010) showed that the six amino acids that differed between males and females at the blastocyst stage no longer differed at the expanded blastocyst stage, whereas two other amino acids did, which shows transient differences between the metabolisms of male and female embryos. The significant sex*stage interaction identified in our study also points to transient differences between male and female embryos. More precisely, our results evidenced a difference linked to embryo sex in the kinetics of metabolite consumption between the expanded and hatched blastocyst stages. For these metabolites, the balance between release and consumption suddenly shifted in favor of consumption among female embryos, while it remained about the same over the developmental period for male embryos.

Eight amino acids displayed significant interactions. Five of these were previously identified as showing differences in the spent culture medium depending on the sex of the embryo, although these studies were conducted under different conditions of embryo development and at different stages, and used different analytical methods: methionine (Sturmey 2010, Gomez et al. 2016, Gimeno et al., 2022), isoleucine (Rubessa et al., 2018), threonine (Gomez et al. 2016, Muñoz et al. 2020, Gimeno et al., 2022), glycine (Muñoz et al. 2020, Sturmey et al., 2010), and valine (Gomez et al. 2016, Rubessa et al., 2018, Gimeno et al., 2022, Sturmey 2010). Isoleucine and valine uptake displayed a significant sex*development period in the analysis of Rubessa et al. (2018) and glycine had an increased depletion in media containing IVD female blastocysts in Sturmey’s study which is consistent with our results. However, other previous results were not fully consistent with ours. For example, methionine followed the same pattern as glycine but only for IVP female embryos and valine was found more depleted by male IVP embryos in Sturmey et al. (2010). These differences are probably due to differences in embryo stages and grade, culture conditions, and methods of quantification. Consequently, the scope of these comparisons is very limited. The transient nature of the differences we observed also contributes to this lack of consistency and previous studies also failed to reach a consensus. Thus, intra-study comparisons are more informative than inter-study comparisons.

Whether the differences we observed had functional consequences on embryo development remains unknown. Interestingly, several amino acids are involved in blastocyst formation (Winkle, 2021), and threonine is necessary for mouse pluripotent stem cell proliferation (Winkle et al., 2014). Thus, subtle differences in amino acid uptake kinetics between male and female blastocysts may induce, or reflect subtle differences in the regulation of blastocyst development. Methionine is necessary for the morula to blastocyst transition in the bovine and for DNA methylation level regulation (Ikeda et al., 2012). During the blastocyst stage, which coincides with the process of X-chromosome inactivation in female embryos (Yu et al., 2020), a sudden increase in methionine uptake may be essential for female embryos.

On the other hand, alanine regulates pH and participates to detoxification of ammonia groups produced by amino acid metabolism in bovine blastocyst (Orsi and Leese 2004). It is among the most abundant amino acids in the bovine blastocoele, and is produced by both the inner cell mass and the trophectoderm (Gopichandran & Leese, 2003), so that subtle differences in its uptake kinetics may have little or no consequences on embryo physiology. Whether the differences in its uptake between male and female blastocysts result from differential expression or function of its transporter remains to be investigated. Indeed, branched amino acids: leucine, isoleucine and valine shared with alanine the pattern of sex*Day-7 stage interaction, and the same B^0,+^ transport system in mammalian blastocyst (Winckle et al. 2006). Glycine is also a very abundant amino acid in the reproductive tract, but is involved in bovine blastocyst hatching (Herrick et al., 2016). A sex-dimorphism in its uptake between the expanded and hatched blastocyst stages could thus reveal differences in the regulation of this step which is necessary for further development.

## Conclusion

Our experimental design evidenced a significant decrease in inter-individual variability in embryo metabolic activity among IVP embryos compared to their IVD counterparts. The reduced variability of IVP embryos, partly dependent on IVP conditions, could be due to the absence of embryo-maternal dialogue on the one hand, and to standardization through group culture on the other.

It invites new questions regarding the impact of embryo biotechnology. While research continues in terms of their effects on embryo quality, their impact on the differences that exist in vivo between embryos is not. It is legitimate to ask whether such standardization is beneficial when embryos will be transferred into physiologically different recipients.

The decrease in variability is such that analyzing the impact of an embryo’s biological feature on spent culture medium no longer appears relevant for these IVP embryos. However, our results have highlighted the dynamic use of metabolites from the culture medium by IVD embryos. This dynamic is consistent with our knowledge of the increased needs of embryos from the morula-blastocyst stage. Moreover, during the blastocyst stage and for some metabolites, this use depends transiently on the sex of the embryo. The functional consequences of which remain to be investigated, and may vary with metabolites. The highly transient nature of this difference between male and female embryos will make it difficult to define environments that could favor the preferential development of embryos of a given sex.

## Supplementary Information

Below is the link to the electronic supplementary material.

Supplementary Material 1

## Data Availability

data are available at [https://entrepot.recherche.data.gouv.fr/dataset.xhtml? persistentId=doi:10.57745/W5BYJ3](https:/entrepot.recherche.data.gouv.fr/dataset.xhtml? persistentId=doi:10.57745/W5BYJ3).
