# Supplementary material for: In vitro production significantly reduces metabolic differences among bovine embryos
Source: Metabolomics. 2025 Dec 13;22(1):12. doi: 10.1007/s11306-025-02352-x (PMC12701867; doi:10.1007/s11306-025-02352-x)
Supplement: Supplementary file 1 — Supplementary Material 1 [file 11306_2025_2352_MOESM1_ESM.docx]

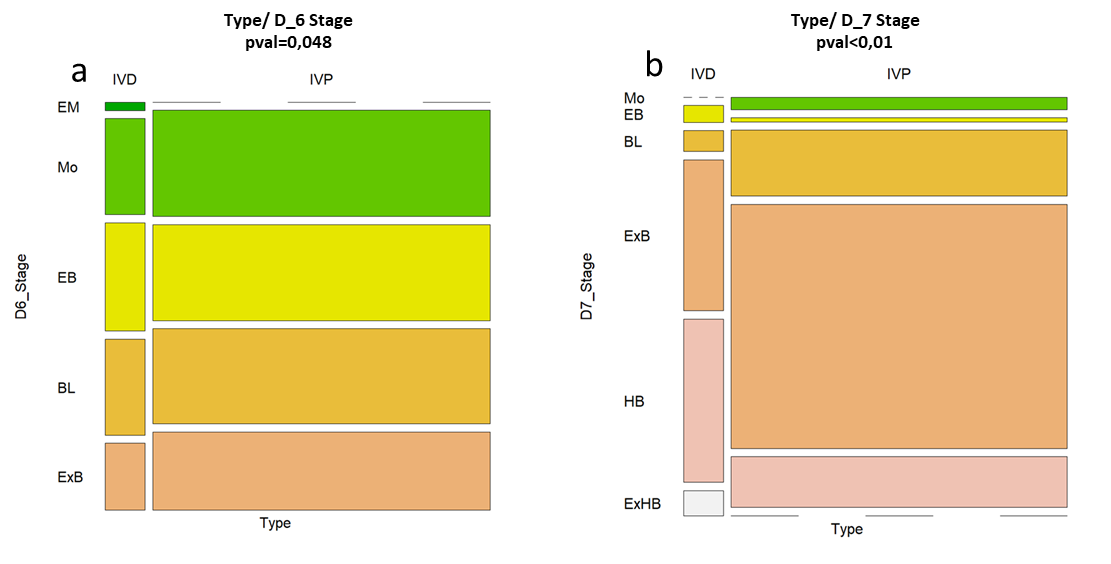


c

IVD

IVP


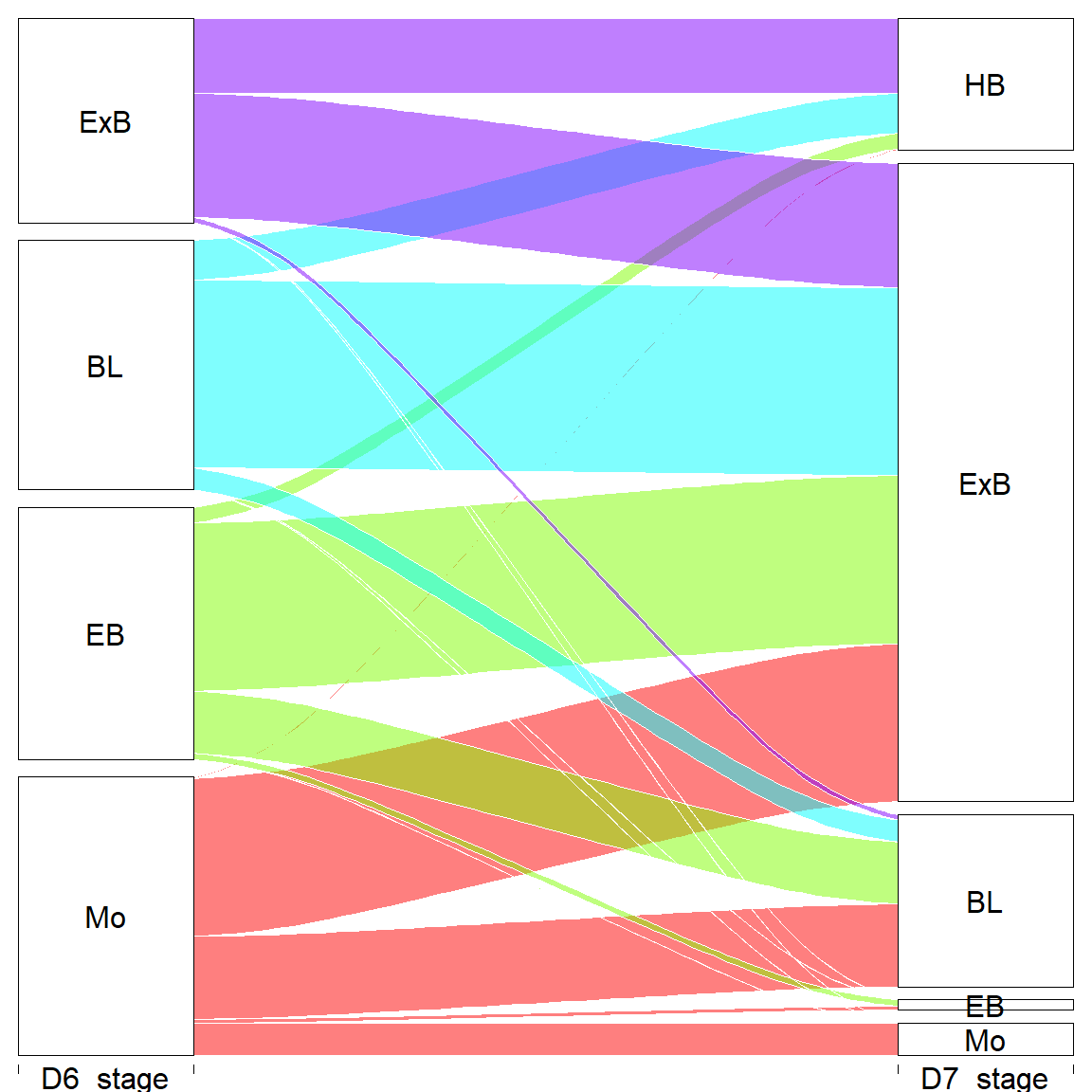

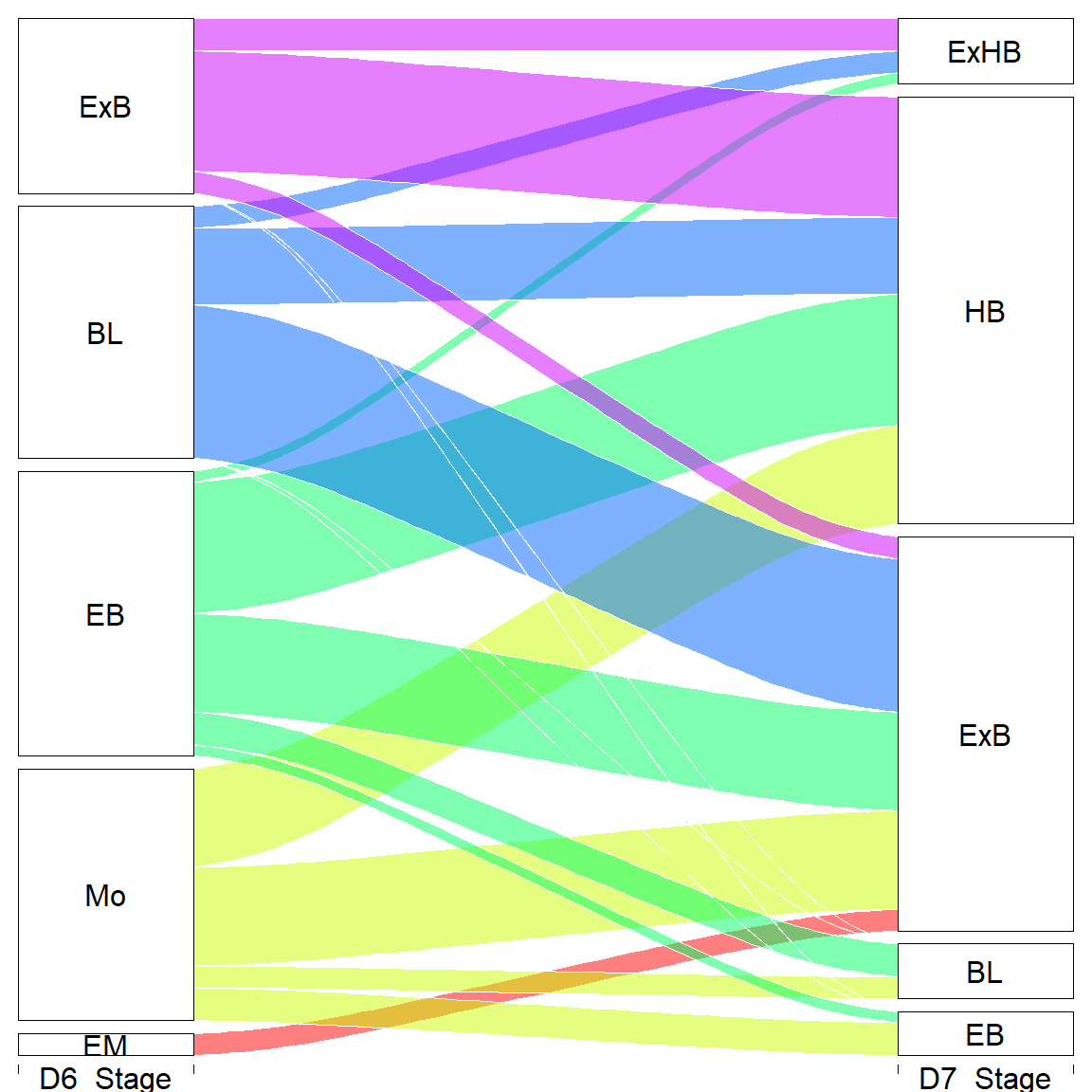


Supplementary Fig1. Mosaic plots comparing IVD and IVP embryos’ stages at beginning (Day-6) (a) and end (Day-7) (b) of the single embryo culture period. (c) Alluvial plots displaying embryos’ stage evolution during the single embryo culture period for in vivo developed (IVD) and in vitro produced (IVP) embryos. Each color corresponds to a specific stage indicated on the vertical axis.

(EM : Early morula, Mo :Morula, EB : Early Blastocyst, BL : Blastocyst, ExB : Expanded Blastocyst, HB : Hatched Blastocyst, ExHB : expanded hatched blastocyst).


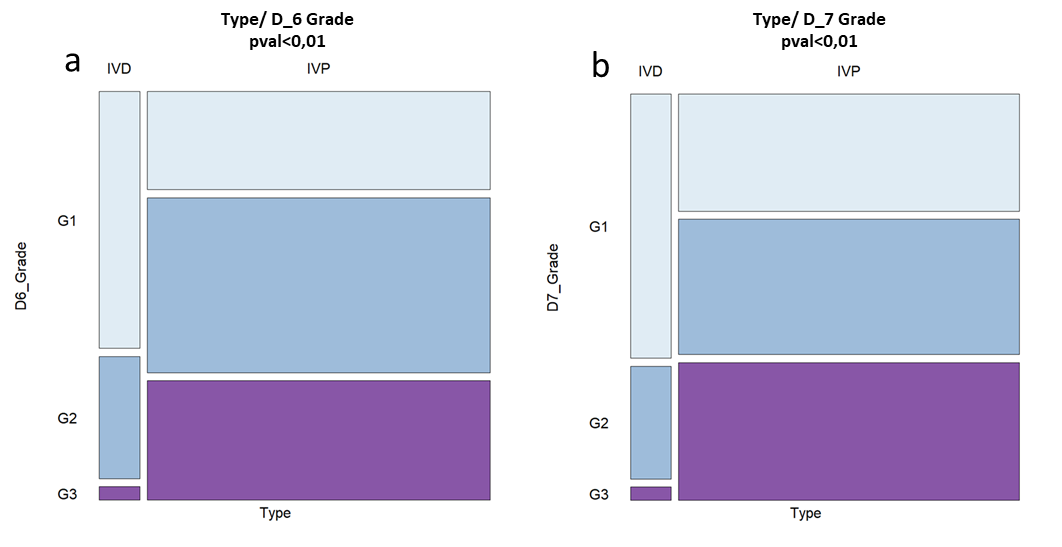


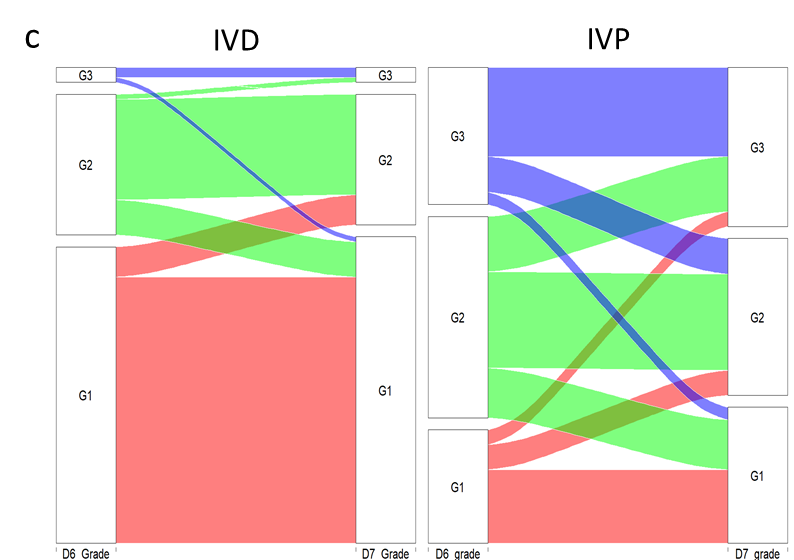


Supplementary Fig2. Mosaic plots comparing IVD and IVP embryos’ grades at beginning (Day-6) (a) and end (Day-7) (b) of the single embryo culture period. (c) Alluvial plots displaying embryos’ grade evolution during the single embryo culture period for in vivo developed (IVD) and in vitro produced (IVP) embryos. Each color corresponds to a specific grade indicated on the vertical axis.

(G1, G2, G3: Grade 1,2,3)

Supplementary Fig3. Multiple Correspondence Analysis of in vivo developed (a) and in vitro produced (b) embryos stage and grade data. X-axis and Y-axis show the first and second components with the percentage of explained variance respectively. The same factorial plane (Axes 1 & 2) is plotted four times, points are colored according to embryo’s Day-6 stage (top left), Day-6 grade (top right), Day-7 stage (bottom left) and Day-7 grade (bottom right).

(Mo: Morula, EB: Early Blastocyst, BL: Blastocyst, ExB: Expanded Blastocyst, HB: Hatched Blastocyst, ExHB: Expanded Hatched Blastocyst, G1, G2, G3: Grade 1,2,3)


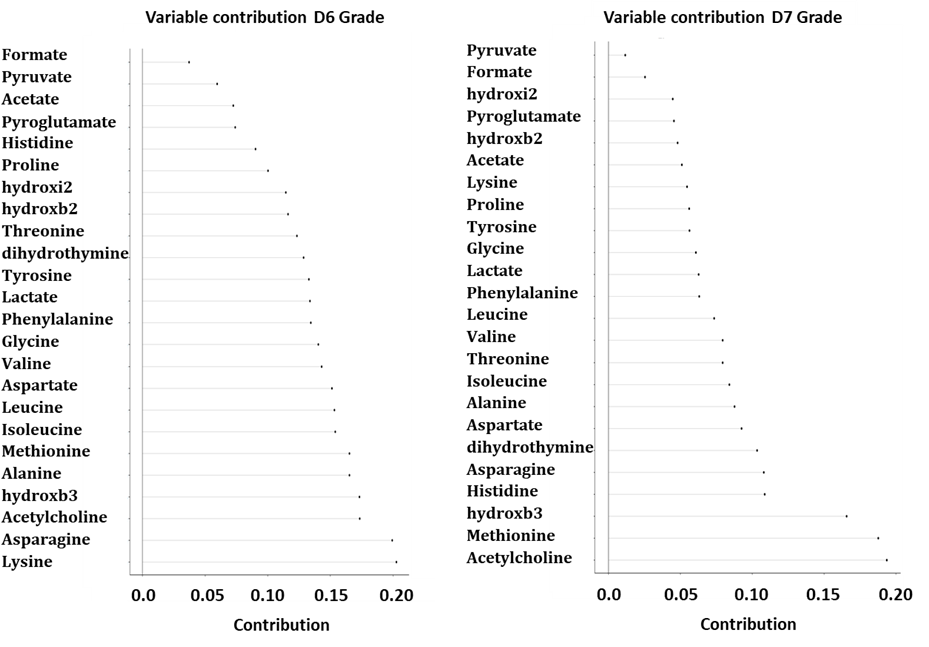

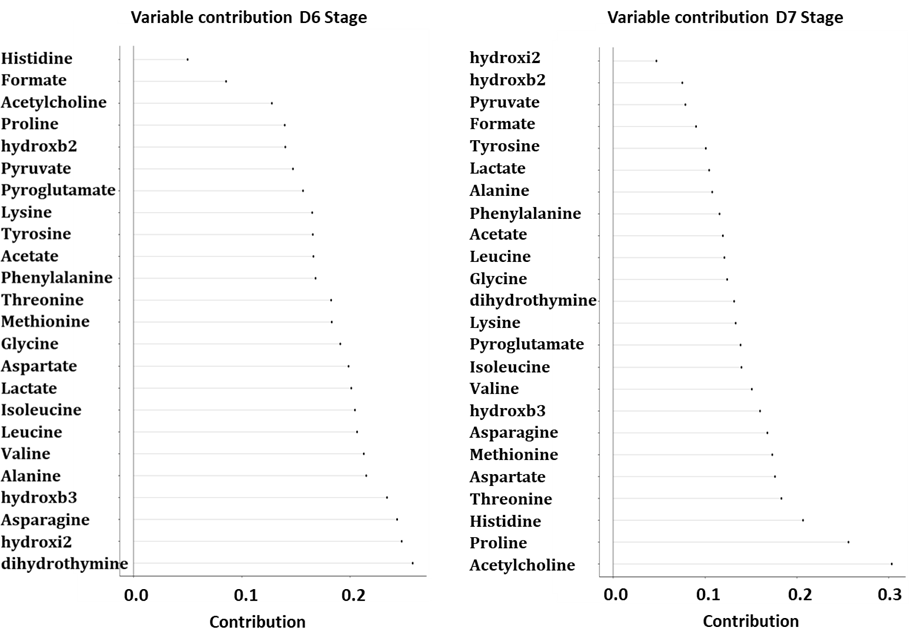


Supplementary Fig4. Representation of metabolites’ contributions to Stage (a,b) and Grade (c,d) at Day-6 (a,c) and Day-7 (b,d).

(hydroxb2: 2-hydroxybutyrate, hydroxi2: 2-hydroxyisobutyrate, hydroxb3: 3-hydroxybutyrate, dihydrothymine: 5,6-dihydrothymine).


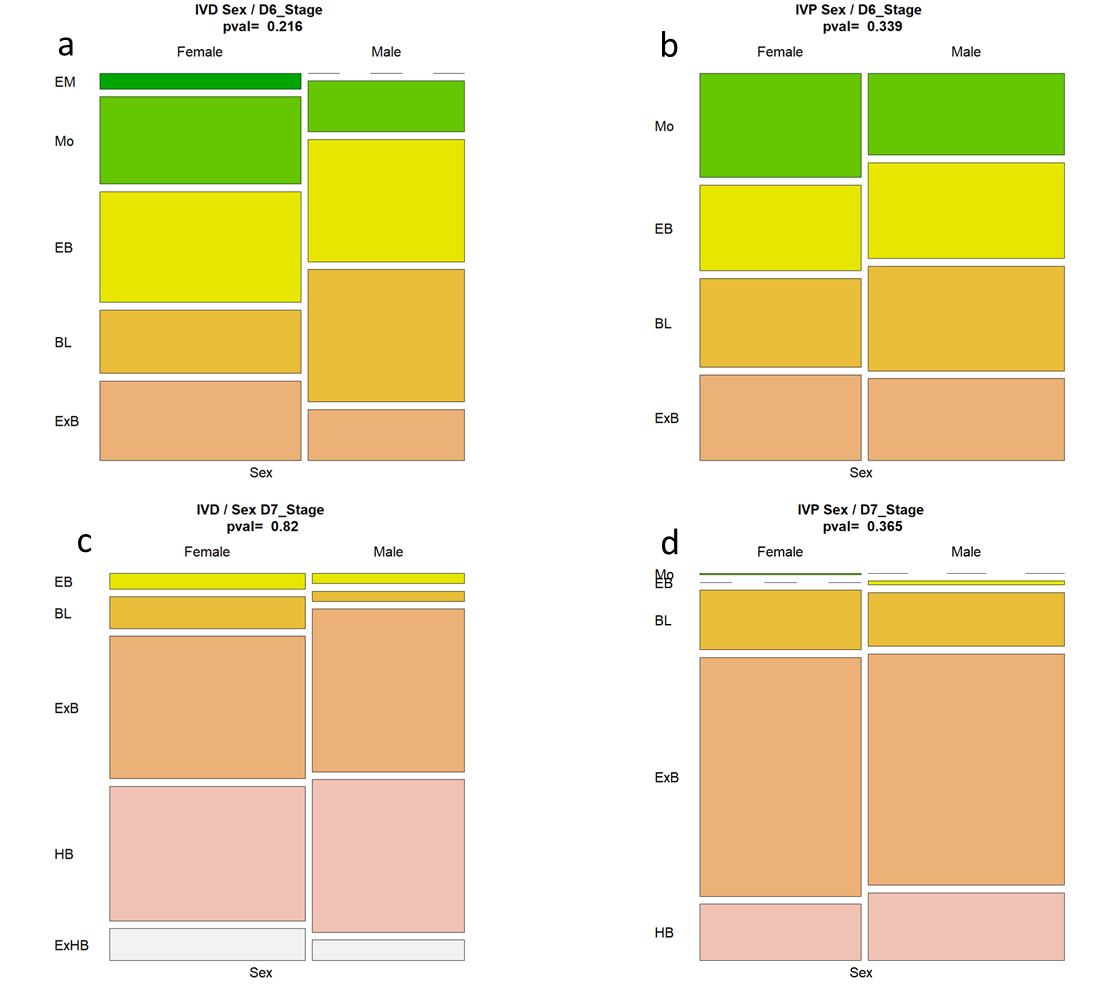


Supplementary Fig5. Mosaic plots of the distribution of embryos’ stages at Day-6 (a,b) and Day-7 (c,d), for IVD (a,c) and IVP (b,d) embryos, according to their sex. Each color corresponds to a specific stage indicated on the vertical axis.

(EM: Early morula, Mo: Morula, EB: Early Blastocyst, BL: Blastocyst, ExB: Expanded Blastocyst, HB: Hatched Blastocyst, ExHB: Expanded Hatched Blastocyst).


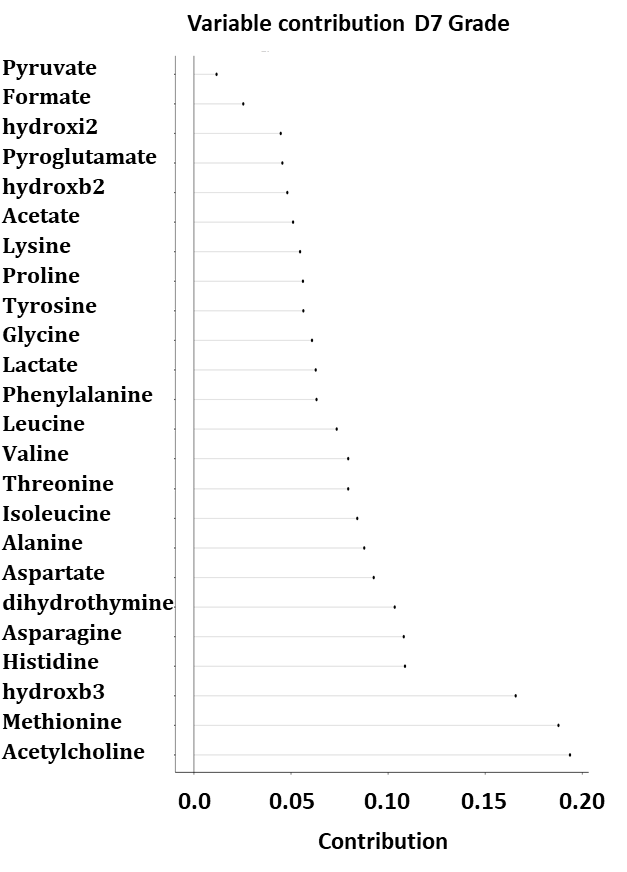


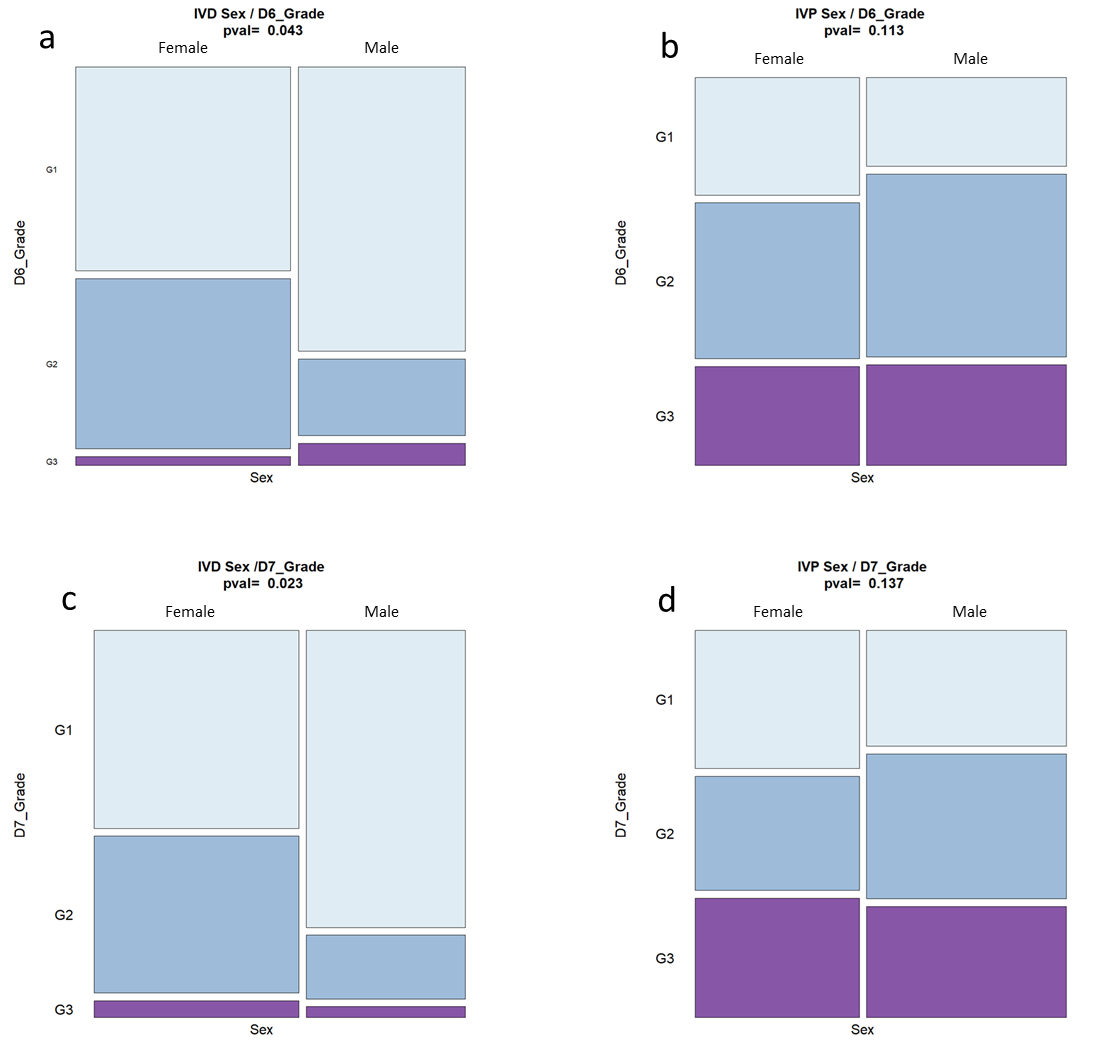


Supplementary Fig6. Mosaic plots of the distribution of IVD (a,c) and IVP (b,d) embryos grades (c,d), for IVD (a,c) and IVP (b,d) embryos, according to their sex. Each color corresponds to a specific grade indicated on the vertical axis.

(G1, G2, G3: Grade 1,2,3)


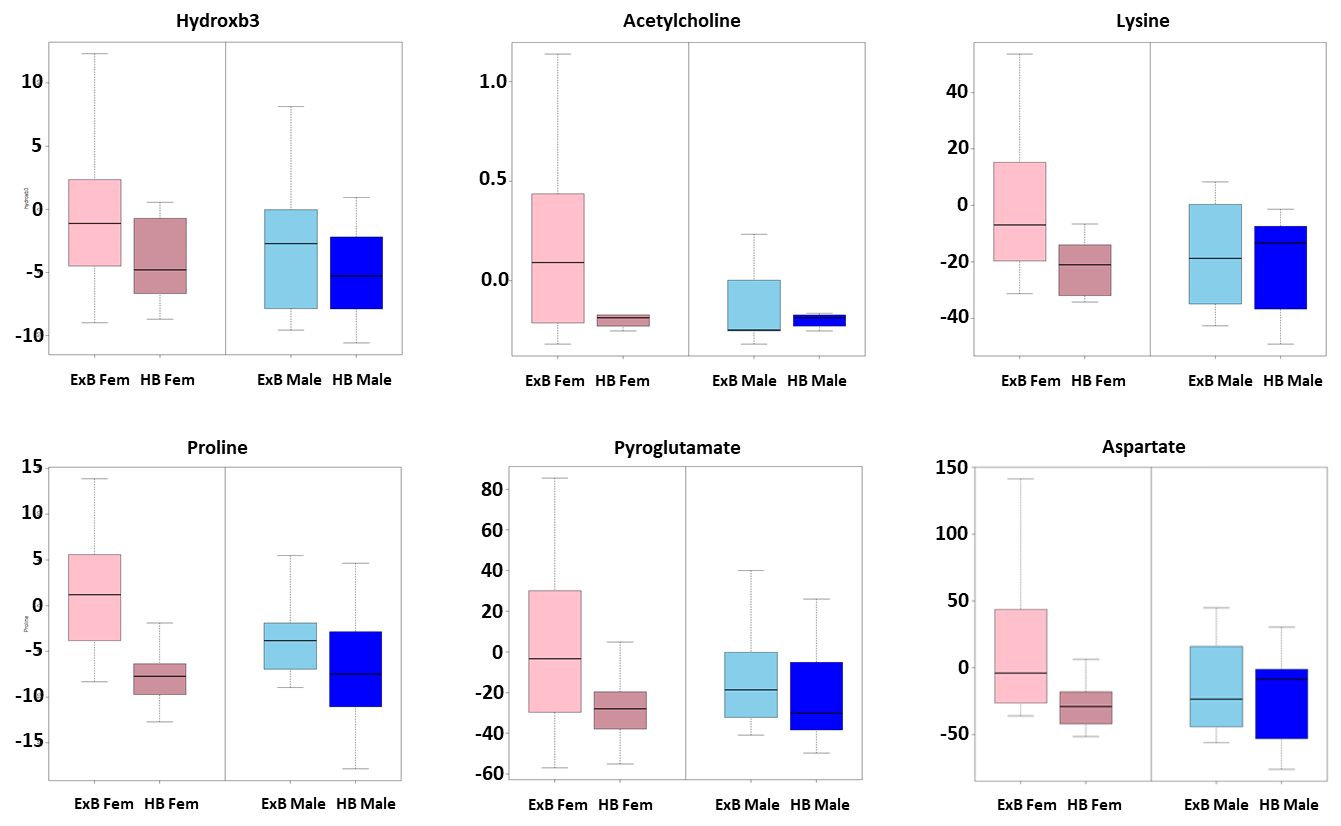


Supplementary Fig7. Quantification of metabolites without significant Sex*Day-7 stage interaction but with a significant (p<0.05) Day-7 stage effect in IVD embryos spent culture media of Female (pink) or Male (blue) embryos (See Sup Table 5). For each metabolite and for each embryonic Day-7 stage and sex plotted on the X-axis, the net balance of extracellular metabolites released or consumed by embryos is plotted on the Y-axis

(hydroxb3: 3-hydroxybutyrate. ExB: Expanded Blastocyst, HB: Hatched Blastocyst. Fem: Female)


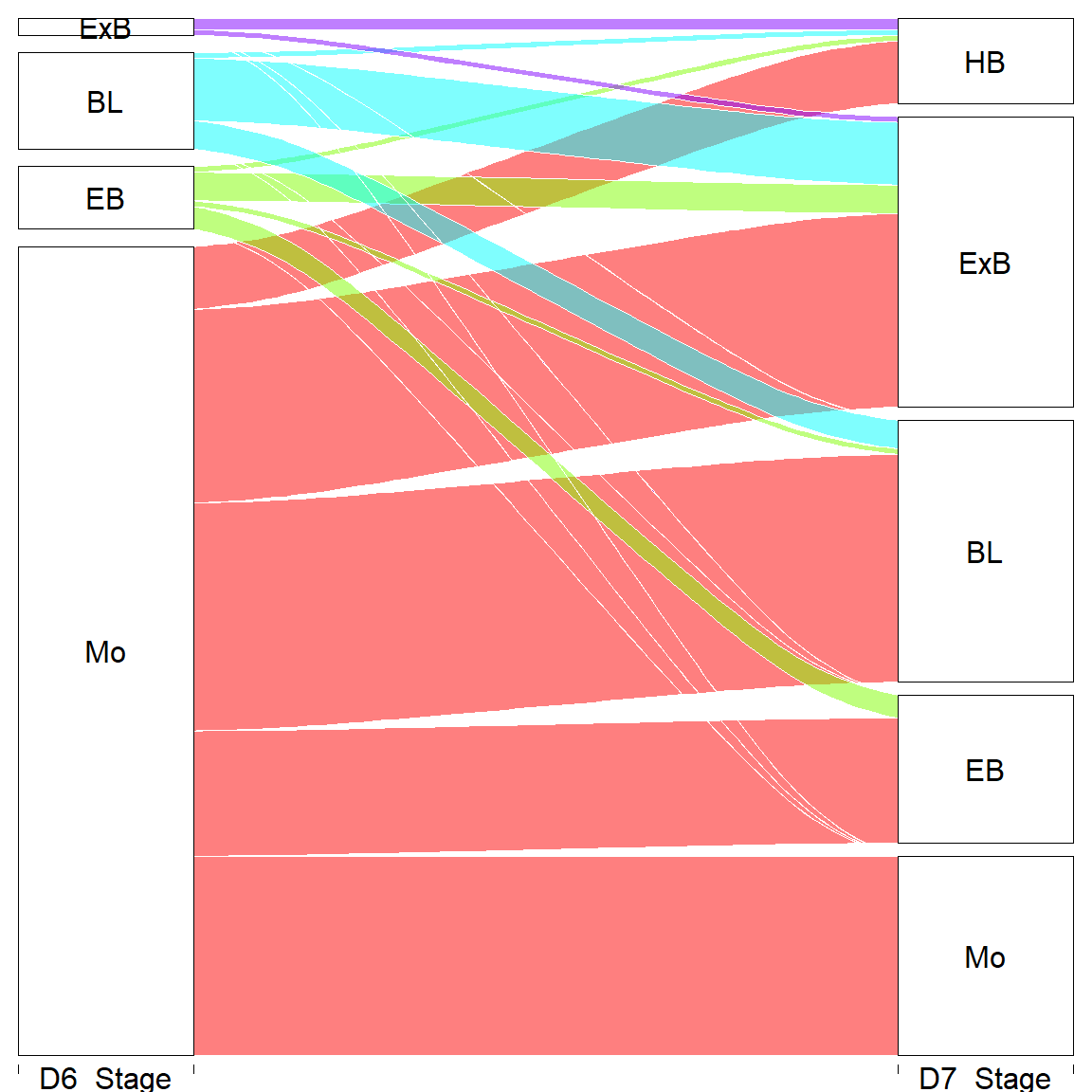


Supplementary Fig8. Alluvial plots displaying embryos’ stage evolution during the single embryo culture period for in vitro produced (IVP) embryos in IVF Biosciences media. Each color corresponds to a specific stage indicated on the vertical axis.

(Mo :Morula, EB : Early Blastocyst, BL : Blastocyst, ExB : Expanded Blastocyst, HB : Hatched Blastocyst, ExHB : expanded hatched blastocyst).

Suppl Table 1a : Numbers (Percentages in brackets ) of IVD and IVP embryos at different stages at Day-6 and Day-7.

(EM: Early morula, Mo: Morula, EB: Early Blastocyst, BL: Blastocyst, ExB: Expanded Blastocyst, HB: Hatched Blastocyst, ExHB: expanded hatched blastocyst).

|  | **IVD** | | **IVP** | |
| --- | --- | --- | --- | --- |
| **Stage** | **Day 6** | **Day 7** | **Day6** | **Day7** |
| **EM** | 2 (2.2) | 0 (0) | 0 | 0 |
| **Mo** | 23 (25.6) | 0 (0) | 215 (28.3) | 25 (3.3) |
| **EB** | 26 (28.9) | 4 (4.4) | 195 (25.6) | 8 (1.1) |
| **BL** | 23 (25.6) | 5 (5.6) | 193 (25.4) | 133 (17.5) |
| **ExB** | 16 (17.8) | 36 (40.0) | 158 (20.8) | 493 (64.8) |
| **HB** | 0 | 39 (43.3) | 0 | 102 (13.4) |
| **ExHB** | 0 | 6 (6.7) | 0 | 0 |
| **TOTAL** | **90** | **90** | **761** | **761** |

Suppl Table 1b : Numbers (Percentages in brackets) of IVD and IVP embryos of different grades at Day-6 and Day-7.

(G1, G2, G3: Grade 1,2,3)

|  | **IVD** | | **IVP** | |
| --- | --- | --- | --- | --- |
| **Grade** | **Day 6** | **Day 7** | **Day6** | **Day7** |
| **G1** | 59 (65.6) | 61 (67.8) | 191 (25.1) | 229 (30.1) |
| **G2** | 28 (31.1) | 26 (28.9) | 339 (44.5) | 264 (34.7) |
| **G3** | 3 (3.3) | 3 (3.3) | 231 (30.3) | 268 (35.2) |
| **TOTAL** | **90** | **90** | **761** | **761** |

Suppl Table 2 : Association between embryos sex and stages or grades. Fisher’s exact test p-values.

| **Type** | **Variable** | **p-value** |
| --- | --- | --- |
| **IVD** | D6_Stage | 0.216 |
| **IVD** | D6_Grade | 0.043 |
| **IVD** | D7_Stage | 0.816 |
| **IVD** | D7_Grade | 0.025 |
| **IVP** | D6_Stage | 0.207 |
| **IVP** | D6_Grade | 0.109 |
| **IVP** | D7_Stage | 0.364 |
| **IVP** | D7_Grade | 0.134 |
| **Both** | D6_Stage | 0.192 |
| **Both** | D6_Grade | 0.319 |
| **Both** | D7_Stage | 0.727 |
| **Both** | D7_Grade | 0.586 |

Suppl Table 3. Redundancy analyses of the IVP and IVD embryos’spent culture media metabolomes modelled according to Sex, Stage or Sex, Grade and their interactions at Day-6 and Day-7. Analyses of variance (test based on 999 permutations; Df : degrees of freedom).

|  | *IVP* | | | | *IVD* | | | |
| --- | --- | --- | --- | --- | --- | --- | --- | --- |
| **Day-6 Grade** | Df | Variance | F | Pr(>F) | Df | Variance | F | Pr(>F) |
| Sex | 1 | 0.015 | 0.336 | 0.966 | 1 | 0.365 | 1.303 | 0.239 |
| D6-Grade | 2 | 0.057 | 0.641 | 0.795 | 1 | 3.039 | 10.852 | 0.001 |
| Sex*D6-Grade | 2 | 0.127 | 1.428 | 0.133 | 1 | 0.156 | 0.558 | 0.569 |
| Residual | 534 | 23.801 | NA | NA | 73 | 20.440 | NA | NA |
|  |  |  |  |  |  |  |  |  |
| **Day-7 Grade** | Df | Variance | F | Pr(>F) | Df | Variance | F | Pr(>F) |
| Sex | 1 | 0.027 | 0.582 | 0.775 | 1 | 0.384 | 1.303 | 0.256 |
| D7-Grade | 2 | 0.109 | 1.179 | 0.252 | 1 | 2.062 | 7.003 | 0.005 |
| Sex*D7-Grade | 2 | 0.124 | 1.343 | 0.170 | 1 | 0.350 | 1.190 | 0.291 |
| Residual | 513 | 23.740 | NA | NA | 72 | 21.203 | NA | NA |
|  |  |  |  |  |  |  |  |  |
| **Day-6 Stage** | Df | Variance | F | Pr(>F) | Df | Variance | F | Pr(>F) |
| Sex | 1 | 0.015 | 0.336 | 0.976 | 1 | 0.325 | 1.205 | 0.257 |
| D6-Stage | 3 | 0.160 | 1.196 | 0.213 | 3 | 3.949 | 4.875 | 0.001 |
| Sex*D6-Stage | 3 | 0.117 | 0.876 | 0.562 | 3 | 0.826 | 1.020 | 0.391 |
| Residual | 532 | 23.708 | NA | NA | 70 | 18.900 | NA | NA |
|  |  |  |  |  |  |  |  |  |
| **Day-7 Stage** | Df | Variance | F | Pr(>F) | Df | Variance | F | Pr(>F) |
| Sex | 1 | 0.059 | 1.066 | 0.336 | 1 | 0.742 | 2.322 | 0.093 |
| D7-Stage | 1 | 0.099 | 1.782 | 0.082 | 1 | 1.904 | 5.958 | 0.005 |
| Sex*D7-Stage | 1 | 0.071 | 1.282 | 0.221 | 1 | 1.537 | 4.809 | 0.014 |
| Residual | 429 | 23.771 | NA | NA | 62 | 19.816 | NA | NA |

Suppl. Table 4a. P-values issued from the analyses of variance of metabolites in IVD embryos’ spent culture media according to the model: Metabolite=Sex+Day6-Stage + (Sex*Day6-Stage).

| **Metabolite** | **Sex** | **Day6-Stage** | **Sex*Day6-Stage** |
| --- | --- | --- | --- |
| hydroxb2 | 0.863 | 0.032 | 0.989 |
| hydroxi2 | 0.400 | <0.001 | 0.121 |
| hydroxb3 | 0.843 | 0.001 | 1.000 |
| dihydrothymine | 0.638 | <0.001 | 0.900 |
| Acetate | 0.291 | 0.010 | 0.055 |
| Acetylcholine | 0.212 | 0.042 | 1.000 |
| Alanine | 0.0800 | <0.001 | 0.223 |
| Asparagine | 0.441 | <0.001 | 0.672 |
| Aspartate | 0.824 | 0.004 | 0.645 |
| Formate | 0.667 | 0.063 | 0.114 |
| Glycine | 0.072 | 0.006 | 0.157 |
| Histidine | 0.619 | 0.084 | 0.053 |
| Isoleucine | 0.082 | 0.006 | 0.368 |
| Lactate | 0.068 | 0.007 | 0.351 |
| Leucine | 0.050 | <0.001 | 0.319 |
| Lysine | 0.363 | 0.003 | 0.654 |
| Methionine | 1.000 | 0.022 | 1.000 |
| Phenylalanine | 1.000 | 0.001 | 0.922 |
| Proline | 0.191 | 0.004 | 0.238 |
| Pyroglutamate | 0.486 | 0.007 | 0.413 |
| Pyruvate | 0.117 | 0.037 | 0.769 |
| Threonine | 0.615 | 0.003 | 0.589 |
| Tyrosine | 0.347 | 0.002 | 0.740 |
| Valine | 0.226 | 0.004 | 0.324 |

hydroxb2: 2-hydroxybutyrate, hydroxi2: 2-hydroxyisobutyrate, hydroxb3: 3-hydroxybutyrate, dihydrothymine: 5,6-dihydrothymine.

Suppl Table 4b. Results of the ANOVA test for linear trend for Day-6 Stage for each metabolite.

| **Metabolite** | **Estimate** | **p-val** |
| --- | --- | --- |
| hydroxb2 | -10.18 | 0.006 |
| hydroxi2 | -6.65 | <0.001 |
| hydroxb3 | -5.46 | <0.001 |
| dihydrothymine | -1.15 | <0.001 |
| Acetate | -35.21 | 0.001 |
| Acetylcholine | -0.27 | 0.007 |
| Alanine | -10.43 | <0.001 |
| Asparagine | -86.68 | <0.001 |
| Aspartate | -39.73 | <0.001 |
| Formate | -37.87 | 0.009 |
| Glycine | -32.41 | <0.001 |
| Histidine | -7.37 | 0.013 |
| Isoleucine | -10.37 | <0.001 |
| Lactate | -110.24 | <0.001 |
| Leucine | -8.96 | <0.001 |
| Lysine | -21.03 | <0.001 |
| Methionine | -8.55 | 0.003 |
| Phenylalanine | -10.37 | <0.001 |
| Proline | -5.22 | 0.001 |
| Pyroglutamate | -30.56 | 0.001 |
| Pyruvate | -188.37 | 0.015 |
| Threonine | -20.61 | <0.001 |
| Tyrosine | -9.025 | <0.001 |
| Valine | -18.15 | <0.001 |

hydroxb2: 2-hydroxybutyrate, hydroxi2: 2-hydroxyisobutyrate, hydroxb3: 3-hydroxybutyrate, dihydrothymine: 5,6-dihydrothymine.

Suppl Table 5. P-values issued from the analyses of variance of metabolites in IVD embryos’ spent culture media according to the model : Metabolite=Sex+Day7-Stage + (Sex*Day7- Stage).

| **Metabolite** | **Sex** | **Day7-Stage** | **Sex*Day7-Stage** |
| --- | --- | --- | --- |
| hydroxb2 | 0.745 | 0.725 | 0.490 |
| hydroxi2 | 0.638 | 0.638 | 0.073 |
| hydroxb3 | 0.112 | 0.031 | 0.382 |
| dihydrothymine | 0.269 | 0.418 | 0.603 |
| Acetate | 0.133 | 0.044 | 0.035 |
| Acetylcholine | 0.052 | 0.005 | 0.340 |
| Alanine | 0.040 | 0.059 | 0.006 |
| Asparagine | 0.200 | 0.090 | 0.044 |
| Aspartate | 0.941 | 0.008 | 0.054 |
| Formate | 0.882 | 0.044 | 0.045 |
| Glycine | 0.217 | 0.043 | 0.005 |
| Histidine | 0.505 | 0.151 | 1.000 |
| Isoleucine | 0.057 | 0.027 | 0.016 |
| Lactate | 0.043 | 0.067 | 0.016 |
| Leucine | 0.111 | 0.060 | 0.038 |
| Lysine | 0.633 | 0.012 | 0.902 |
| Methionine | 0.304 | 0.021 | 0.022 |
| Phenylalanine | 0.941 | 0.413 | 0.052 |
| Proline | 0.134 | <0.001 | 0.064 |
| Pyroglutamate | 0.882 | 0.031 | 0.103 |
| Pyruvate | 0.216 | 0.123 | 0.175 |
| Threonine | 0.882 | 0.006 | 0.031 |
| Tyrosine | 0.667 | 0.195 | 0.054 |
| Valine | 0.148 | 0.006 | 0.045 |

hydroxb2: 2-hydroxybutyrate, hydroxi2: 2-hydroxyisobutyrate, hydroxb3: 3-hydroxybutyrate, dihydrothymine: 5,6-dihydrothymine.

Suppl Table 6 a: Numbers (Percentages in brackets) of IVP embryos produced in IVF Bioscience media at different stages at Day-6 or Day-7.

(Mo: Morula, EB: Early Blastocyst, BL: Blastocyst, ExB: Expanded Blastocyst, HB: Hatched Blastocyst).

| **Stage** | **Day-6** | **Day-7** |
| --- | --- | --- |
| **Mo** | 142 (83) | 35 (20.2) |
| **EB** | 11 (6.4) | 26 (15.0) |
| **BL** | 17 (9.8) | 46 (26.6) |
| **ExB** | 3 (1.7) | 51 (29.5) |
| **HB** | 0 | 15 (8.7) |
| **TOTAL** | 173 | 173 |

Suppl Table 6 b: Numbers (Percentages in brackets) of IVP embryos produced in IVF Bioscience media of different gardes at Day-6 or Day-7.

(G1, G2, G3: Grade 1,2,3).

| **Grade** | **Day-6** | **Day-7** |
| --- | --- | --- |
| **G1** | 105 (60.7) | 142 (82.1) |
| **G2** | 54 (31.2) | 28 (16.2) |
| **G3** | 14 (8.1) | 3 (1.7) |
| **TOTAL** | 173 | 173 |

Suppl Table 7 : Redundancy analyses including Sex, Stage or Sex, Grade and their interactions at

Day-6 and Day-7 for IVP (using IVF Bioscience media) embryos’spent culture media metabolomes.

| ***VITRO - D6-Grade*** | **Df** | **Variance** | **F** | **Pr(>F)** |
| --- | --- | --- | --- | --- |
| Sex | 1 | 0.120 | 0.363 | 0.918 |
| D6-Grade | 1 | 0.424 | 1.281 | 0.252 |
| Sex*D6-Grade | 1 | 0.609 | 1.839 | 0.107 |
| Residual | 69 | 22.847 | NA | NA |
|  |  |  |  |  |
| ***VITRO - D7-Grade*** | **Df** | **Variance** | **F** | **Pr(>F)** |
| Sex | 1 | 0.119 | 0.405 | 0.882 |
| D7-Grade | 1 | 0.310 | 1.052 | 0.323 |
| Sexe*D7-Grade | 1 | 1.778 | 6.039 | 0.011 |
| Residual | 74 | 21.793 | NA | NA |
|  |  |  |  |  |
| ***VITRO - D6-Stage*** | **Df** | **Variance** | **F** | **Pr(>F)** |
| Sex | 1 | 0.104 | 0.334 | 0.929 |
| D6-Stage | 3 | 0.836 | 0.895 | 0.424 |
| Sex*D6-Stage | 2 | 0.324 | 0.519 | 0.788 |
| Residual | 73 | 22.736 | NA | NA |
|  |  |  |  |  |
| ***VITRO - D7-Stage7***  ***(EB-HB)*** | **Df** | **Variance** | **F** | **Pr(>F)** |
| Sex | 1 | 0.436 | 0.694 | 0.624 |
| D7-Stage | 1 | 1.064 | 1.695 | 0.131 |
| Sex*D7-Stage | 1 | 0.537 | 0.855 | 0.475 |
| Residual | 35 | 21.964 | NA | NA |

Suppl Table 8 : Comparison of metabolite variances according to embryo type. In column 2 variance for IVP embryos produced in IFV Bioscience media: (IVP2). In columns 3 and 4 variance ratios between the three conditions. The number of stars indicates the level of statistical significance of the corresponding Levene test for variance homogeneity: *** P<0.01, **P<0.05, * P<0.10; NS: non significant.

| **Metabolite** | **IVP2** | **Ratio IVD/IVP2** | **Ratio IVP2/IVP** |
| --- | --- | --- | --- |
| hydroxb2 | 218.39 | 1.73 (NS) | 2.56 *** |
| hydroxi2 | 5.65 | 7.56 *** | 3.69 *** |
| hydroxb3 | 18.56 | 2.38 *** | 0.01 *** |
| dihydrothymine | 1.31 | 1.22 (NS) | 2.9 *** |
| Acetate | 39.99 | 56.24 *** | 1.99 *** |
| Acetylcholine | 0.18 | 0.95 * | 0.06 *** |
| Alanine | 25.08 | 4.98 *** | 2.33 ** |
| Asparagine | 620.94 | 13.8 *** | 0.48 *** |
| Aspartate | 783.82 | 2.81 *** | 2.84 *** |
| Formate | 93.11 | 42.49 *** | 1.13 *** |
| Glycine | 713.76 | 2.00 *** | 5.14 *** |
| Histidine | 51.48 | 2.70 *** | 0.69 (NS) |
| Isoleucine | 18.64 | 7.51 *** | 1.31 (NS) |
| Lactate | 1763.02 | 9.02 *** | 1.17 ** |
| Leucine | 15.73 | 6.67 *** | 1.88 *** |
| Lysine | 209.14 | 2.95 *** | 3.18 *** |
| Methionine | 27.99 | 5.17 *** | 0.99 (NS) |
| Phenylalanine | 43.97 | 3.33 *** | 2.08 *** |
| Proline | 46.95 | 0.90 (NS) | 2.47 *** |
| Pyroglutamate | 1538.16 | 1.02 (NS) | 5.22 *** |
| Pyruvate | 17326.28 | 7.62 *** | 0.28 ** |
| Threonine | 686.03 | 0.86 (NS) | 3.88 *** |
| Tyrosine | 58.92 | 2.19 *** | 3.28 *** |
| Valine | 78.11 | 5.23 *** | 1.05 (NS) |
| Mean | 1016.05 | 7,97 | 2,11 |

hydroxb2: 2-hydroxybutyrate, hydroxi2: 2-hydroxyisobutyrate, hydroxb3: 3-hydroxybutyrate, dihydrothymine: 5,6-dihydrothymine
